# Interpretable prediction of zinc ion location in proteins with ZincSight

**DOI:** 10.1002/pro.70350

**Published:** 2025-10-30

**Authors:** Gilad Mechtinger, Gabriel Axel, Rachel Kolodny, Nir Ben‐Tal

**Affiliations:** ^1^ School of Neurobiology, Biochemistry and Biophysics, George S. Wise Faculty of Life Sciences Tel Aviv University Ramat Aviv Israel; ^2^ Department of Computer Science University of Haifa Haifa Israel

**Keywords:** binding site, binding site prediction, metal ion, metalloprotein, protein function annotation, transition metal ion, zinc ion

## Abstract

Metal ions are essential for a broad range of biochemical processes in living organisms, with zinc being the second most abundant transition metal ion. Zinc has catalytic, structural, and regulatory functions in proteins, impacting virtually all aspects of cell biology. Currently, there are notable challenges in performing a large‐scale accurate systematic analysis of the as‐yet unexplored occurrences of zinc ion in nature. To address this, we developed ZincSight for predicting zinc‐binding sites. ZincSight performs on par with existing structure‐based tools in terms of the precision‐recall curve for zinc ion detection, and the accuracy of spatial positioning of the ion, yet it is significantly faster, and offers a straightforward reasoning for its predictions, which is missing even in the best alternatives. Tests using a panel of metals show that, while trained on zinc‐binding sites, ZincSight in fact detects all transition metal binding sites alike – a reflection of the similarity in coordination among the transition metals. It also detects binding sites for calcium and other alkaline‐earth metals with lower accuracy, but not alkali metal binding sites. Suitable for exploring the usage of zinc and other transition metals in large sets of protein structures, or models thereof, ZincSight is available as a free‐to‐download open‐source software at: https://github.com/MECHTI1/ZincSight. A Google Colab notebook is available at: https://colab.research.google.com/github/MECHTI1/ZincSight/blob/master/ZincSight.ipynb.

## 1 INTRODUCTION

Metals are versatile cofactors in protein biochemistry with an abundance of distinctive properties such as electron‐acceptor ability, positive charge, flexible coordination sphere, specific ligand affinity, varying valence state, low‐ or high‐spin configuration, and mobility/diffusivity (Dudev & Lim, 2014). These properties render metals effective protein cofactors. Indeed, the many so‐called metalloproteins need to bind one or more metal ions to perform their function. It has been estimated that between 25% and 50% of all proteins found within an organism are metalloproteins (Bowman et al., 2016; Ye et al., 2022; Zhang & Zheng, 2020). The bound metal ions in metalloproteins are involved in important functions such as stabilizing protein structures, enzymatic reactions, and regulatory functions (Andreini et al., 2006; Bowman et al., 2016; Maret, 2010). Zinc is the second most abundant transition metal ion in living organisms (after iron) and is essential for Life (Andreini et al., 2006). Zinc has an integral role in stabilizing protein structures and plays pivotal roles in numerous enzymatic catalysis reactions, spanning all seven classes of enzymes. Additionally, zinc is crucial for a wide range of cellular functions and regulatory processes (Andreini et al., 2006; Maret, 2017; McCall et al., 2000; Zhang & Zheng, 2020).

Currently, there is a substantial knowledge gap regarding metal ion utility due to the limited amount of high‐resolution structures of metalloproteins in the Protein Databank (PDB) (Berman, 2000; Ye et al., 2022; Zhang & Zheng, 2020). Filling this gap by predicting metal ion binding sites for a variety of metal ion types, including zinc, may now be feasible. To date, there are highly reliable structural models for millions of proteins predicted from the genomic sequences of many organisms (Kim et al., 2024; Lin et al., 2023; Nomburg et al., 2024; Varadi et al., 2022, 2024). Among these, the AlphaFold (AF) models are particularly relevant because of their accuracy (Jumper et al., 2021; Varadi et al., 2022, 2024). While predicted AF models do not include metal ion cofactors, they open a door for extensive structural bioinformatics prediction of their metal ion binding sites.

Conducting large‐scale systematic analysis requires accessible, user‐oriented tools that accurately predict zinc ion binding sites at high speed. Available metal ion binding predictors are either sequence‐based or structure‐based. Sequence‐based predictors include—mebipred (Aptekmann et al., 2022), LMetalSite (Yuan et al., 2022), M‐Ionic (Shenoy et al., 2024), MIBPred (Zhang et al., 2024), and MetaLLM (Shishir et al., 2023). The sequence‐based tools do not predict the binding site but rather only the probability per protein and/or residue of metal ion binding, and are not necessarily specialized for zinc. Structure‐based predictors include AlphaFill (Hekkelman et al., 2023), Master of Metals (MoM) (Laveglia et al., 2023), PinMyMetal (PMM) (Zheng et al., 2024), and Metal3D (Dürr et al., 2023). AlphaFill “transplants” into the query structure small molecules and ions that have been experimentally observed in homologous protein structures, which makes the existence of such homologous complexes a limiting factor for the method's applicability. MoM uses machine learning to identify triads or quadruplets of C*α* and C*β* atoms of amino acids with appropriate relative spatial arrangements and ranks them based on structural similarity to a library of zinc ion‐binding site templates. PMM combines machine learning with geometric constraints to predict metal‐ion binding residues and ion locations. It integrates physicochemical properties and geometric rules, including interatomic distances, bond angles, coordination number, amino acid identity, and hydrophobicity to classify zinc‐binding sites. The state‐of‐the‐art Metal3D is a 3D convolutional neural network that demonstrated superior accuracy in identifying zinc ion locations compared to previously available tools. Unfortunately, it is relatively slow, taking typically 25 s to process a protein of 250 residues on a multicore GPU workstation (Dürr et al., 2023), and therefore is less suitable for high‐throughput application.

Here we present ZincSight, a user‐friendly, fast, and accurate tool, suitable for predicting zinc ion‐binding sites, that is, clusters of amino acids with high zinc affinity, that can be applied at a genome‐wide scale. Our hypothesis is that zinc binding is local and determined purely based on stereochemistry. Namely, that a cluster of three or more residues that feature the right functional groups in close proximity to each other in the right orientation is sufficient for zinc binding. By clustering zinc‐binding sites based on their amino acid composition and stereochemistry, we curated a set of templates from protein‐zinc co‐complexes of known structure. To predict a zinc‐binding site, we search for a similar template and if found, the training‐set template is provided to support and explain our prediction. We compare the predictions of our method to the current state‐of‐the‐art Metal3D on five test sets: two used in the Metal3D study, one with high‐resolution zinc‐binding PDB structures, and two curated from AF models. We show that ZincSight is comparable to the state‐of‐the‐art methods, such as Metal3D and PMM, in terms of precision, recall, and location accuracy, while being faster and more easily interpretable. Our examination shows that ZincSight indistinctively detects binding sites of all transition metal ions, not just zinc. This enables the systematic screening of predicted protein structures for transition metal ions‐binding at genomic scale.

## 2 RESULTS

ZincSight identifies candidate zinc‐binding sites by searching for specific residue combinations located within predefined distances from each other. These combinations are the ones in our template library, derived from experimentally solved zinc‐binding sites. Subsequently, ZincSight places a zinc ion in the same location with respect to these candidate sites as in the template and assigns confidence scores to its predictions. The score combines the following features, with calibrated weights (see Methods for details):

- Structural similarity between the candidate zinc‐binding site and an experimentally resolved structural zinc‐binding template, with the same amino acid combination. The structural similarity is quantified by the root mean square deviation (RMSD) of the positions of the corresponding zinc‐binding atoms in the candidate and template sites.
- The deviations from ideal values of the distances from the predicted location of the candidate zinc ion and its binding residues.
- The deviation from ideal values of the spatial orientation of the histidine's imidazole group(s) with respect to the predicted location of the zinc ion when the binding residues include histidine(s)—one of the two most frequent amino acid types that interact with zinc (Laitaoja et al., 2013; Tamames et al., 2007). We incorporate this feature in the score because Tamames et al. showed that in 994 high‐quality protein crystal structures solved at a resolution better than 2.5 Å, histidine residues are involved in 48% of the zinc‐ligand interactions, emphasizing their importance (Hekkelman et al., 2023).

### 2.1 Development and training of ZincSight

We developed and tested ZincSight using the training set of 2085 proteins and test set of 59 proteins previously used by Metal3D (Dürr et al., 2023). This is a 30% sequence non‐redundant set of proteins deposited in the PDB before 3/2021 (see Dürr et al., 2023 for more information). With the training dataset as a starting point, we curated a library of template zinc‐binding sites representing diverse and unique combinations of zinc‐binding residues. To minimize the use of non‐biologically relevant zinc‐binding sites as templates, we selected templates that fulfilled three requirements:

- Zinc ion binding is coordinated by at least three residues. This criterion mitigates the inclusion of artifacts prevalent in sites with fewer than three coordinating ligands (Laitaoja et al., 2013), while capturing the majority of enzyme and structural site coordination spheres (Daniel & Farrell, 2014).
- The residue combination appears in at least three different binding sites within at least three distinct PDB structures in the training dataset. This is intended to avoid combinations that are due to experimental artifacts or chance.
- The zinc ion occupancy within these sites is higher than 0.5.

This selection process yielded 42 unique zinc‐binding structural templates. The 2043 structures in the training set that are not in this list of 42 templates are our validation set, and we use these to calibrate the parameters used in ZincSight's formulas. We selected parameters that maximize the area under the precision‐recall curve (AUC‐PR) in the validation set using the Tree‐structured Parzen Estimator (TPE). Finally, we transformed the raw optimized prediction scores (denoted *s*) into calibrated probabilities (denoted *p*) using Platt Scaling, a logistic regression‐based method, and interpret this probability as confidence in the prediction; see Methods for details. To quantify the reliability of these calibrated probabilities, we computed a Brier score of 0.0346 and a log loss of 0.1163 on an independent validation set (Figure S1c). These low error metrics, together with the clear separation between true and false candidate distributions (Figure S1a,b), suggest the probability calibration is robust.

### 2.2 Performance testing and evaluation

#### 2.2.1 Test datasets

We tested ZincSight on five datasets. The first was curated in the Metal3D study and includes 86 zinc‐binding sites in 59 proteins for which zinc ion positions were experimentally determined. Our dataset includes only the binding sites coordinated by at least three residues in the Metal3D test set, the so‐called “3+” sites. We excluded the 31 zinc‐binding sites in the proteins in this test set that are coordinated by only two unique residues. The second dataset, also curated in the Metal3D study, includes 1183 metal binding sites in 907 proteins (fig. 4 of the Metal3D paper (Dürr et al., 2023)). It covers 11 metal types: The transition metals zinc (Zn^2+^; 86 sites in 59 structures), Co^2+^ (36 sites in 30 structures), Cu^2+^ (115 sites in 68 structures), Fe^2+^ (71 sites in 57 structures), Fe^3+^ (134 sites in 100 structures), Mn^2+^ (128 sites in 100 structures) and Ni^2+^ (106 sites in 93 structures); the alkaline earth metals Ca^2+^ (161 sites in 100 structures) and Mg^2+^ (110 sites in 100 structures); and the alkali metals Na^+^ (118 sites in 100 structures) and K^+^ (118 sites in 100 structures). We curated the third test to explore the degree to which the 42‐templates library covers all known zinc‐binding modes. It includes all x‐ray crystal structures from the PDB, determined at ≤2.5 Å resolution with at least one zinc ion and released after March 5, 2021—the cutoff date of the Metal3D test set. Selecting a single representative per UniProt accession resulted in 830 unique entries. The fourth test set includes 27 modeled structures from the AlphaFold Protein Structure Database (AFDB) with 44 zinc‐binding sites. We could evaluate the accuracy of the AF models because the structures of these proteins were experimentally determined and deposited in the PDB after the cutoff date of the AF Monomer v2.0 training set curation (4/2018). Note that AF Monomer v2.0 is the AF model that was used in predicting the structures in AFDB. The set of 27 structures was selected from among those with experimental structures in complex with zinc ions, from different Pfam families, while making sure that they share at most 50% sequence identity both with the AF training set and with each other. Figure S2 shows that superimposing zinc‐binding regions (of the adjacent three‐residue neighbors) from PDB experimental structures onto the corresponding AlphaFold 2 (AF2) model regions yields a mean RMSD of 0.60 Å with a variance of 0.64 Å; most RMSD values are below 1 Å, with only two outliers above 3 Å. These outliers correspond to the binding sites of proteins Q7MVV4 and Q9NXF7, which we retained in our test set to capture the detrimental impact of inaccurate structural predictions on overall performance. In both cases, the zinc‐coordinating residues exhibit low pLDDT scores, underscoring the model's reduced confidence—and hence sensitivity—at metal‐binding sites. The ground truth for this test set comprises the zinc ion positions, copied from the experimental structures to the AF models. The fifth test set includes AF structures of proteins selected from UniRef50 cluster representatives released after the AF2 monomer training cutoff date and used to study the relationship between pLDDT and the accuracy of our predictions.

### 2.3 Evaluation metrics

**FIGURE 1:**
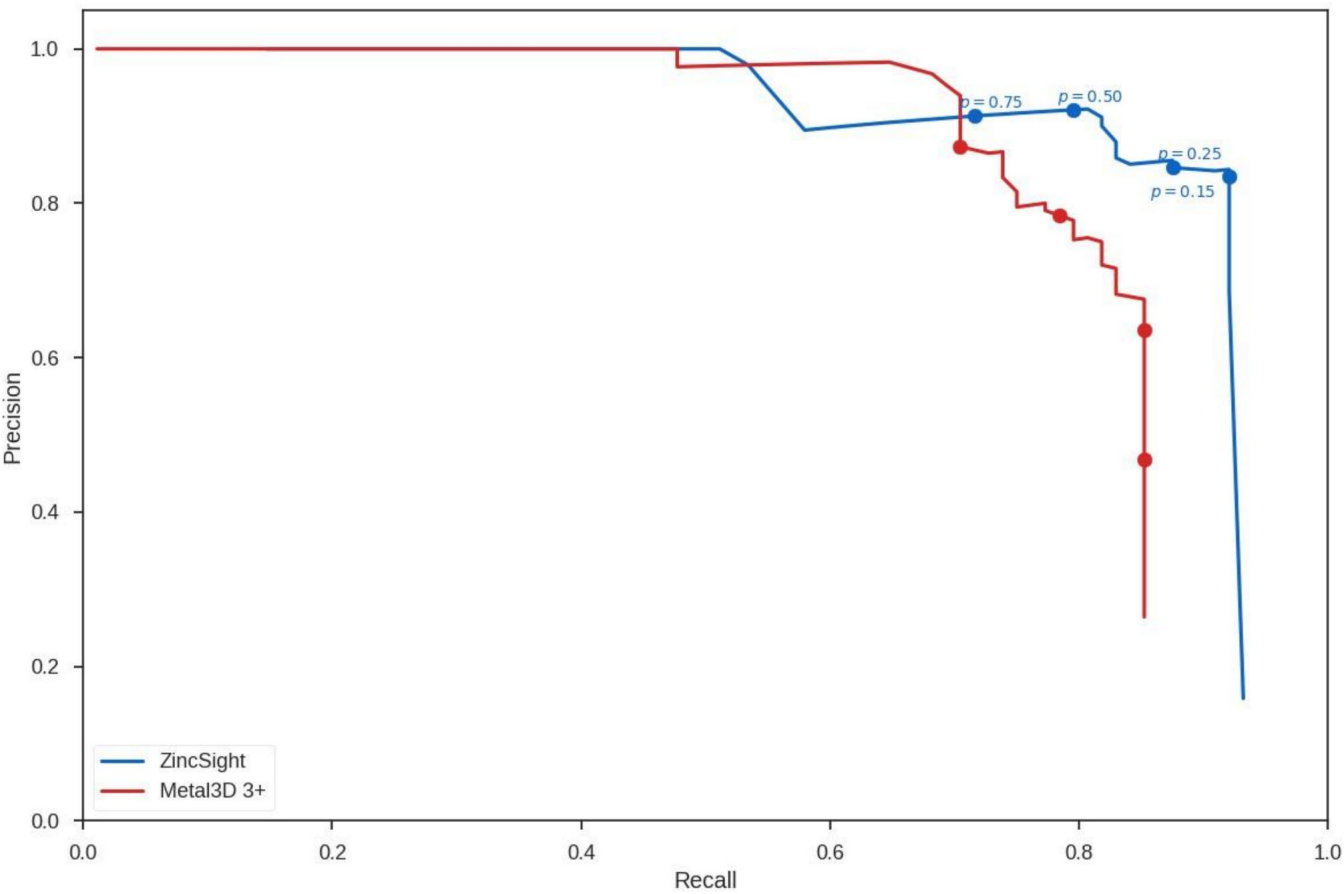
PR in identifying zinc‐binding sites within 2 Å in test set 1. PR for ZincSight (blue curve) and Metal3D (red curve) predictions in test set 1. Test set 1 includes all 86 “3+ zinc sites” of the Metal3D test set of 59 proteins structures. The probability thresholds used for predictions are marked. ZincSight maintains high precision across different recall values. *p* represents the probability that ZincSight assigns to the predictions, indicating its confidence the predicted zinc‐binding sites are to be true.

**FIGURE 2:**
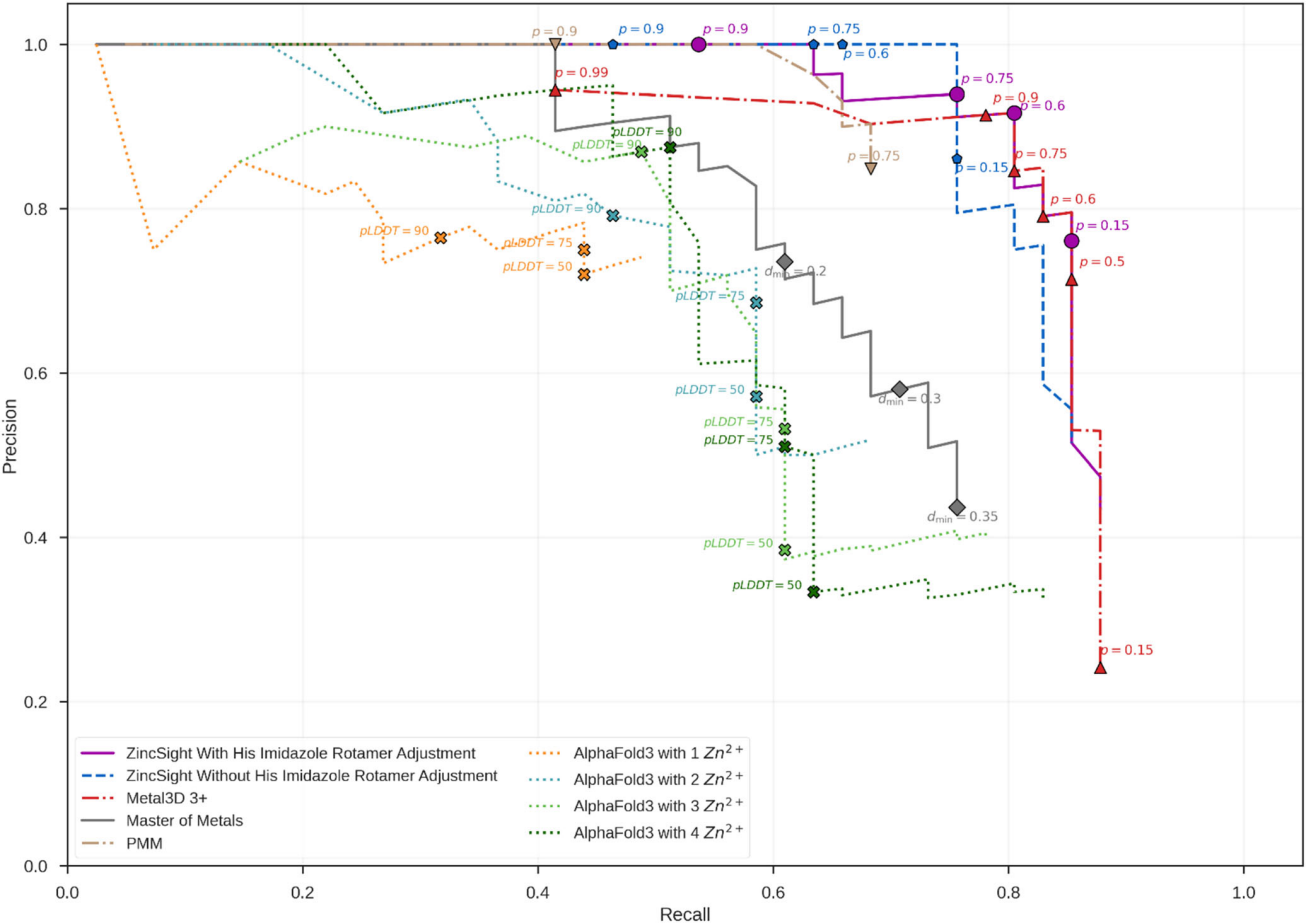
PR in identifying zinc‐binding sites within 2 Å in test set 4. Test set 4 includes 27 modeled structures with 44 zinc‐binding sites from AFDB. The PR curves for ZincSight, Metal3D, MoM, PMM, and AF3 predictions for zinc‐binding sites within this test set. PR metrics for ZincSight predictions were computed for the prediction with (purple curve) and without (blue curve) optimization of the rotameric state of the histidine imidazole. The ground truth zinc ions coordinates were taken from corresponding PDB structures.

**FIGURE 3:**
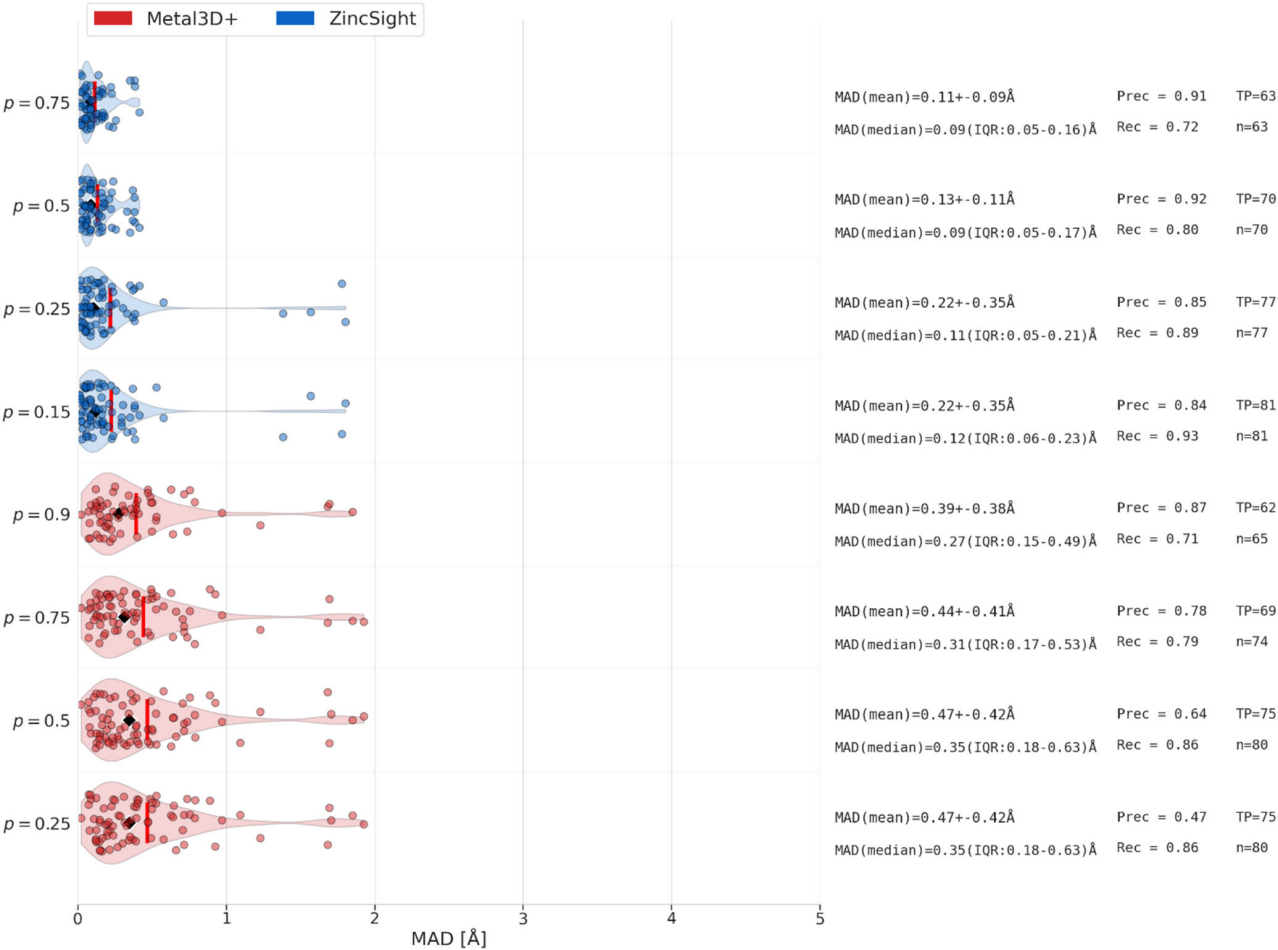
MAD for correctly predicted sites within 2 Å in test set 1. Calculated MAD values for all 86 “3+ zinc sites” in the Metal3D test set that were correctly predicted by ZincSight (blue) and Metal3D (red). ZincSight's MAD values measured at series of predicted probabilities thresholds ranging from 0.75 to 0.15. Per each probability threshold, *n* is the number of the overall predicted metal sites within 2 Å of the experimentally‐determined zinc position. TP is the number of correctly predicted sites (multiple predicted sites can be within 2 Å radius from a single TP site). Each dot represents the measured distance (in Å) between a predicted metal ion position and the experimental zinc ion position. The violin shape shows the kernel density of these distances, with a black vertical line marking the median, a white vertical line marking the mean, and a black horizontal line extended from the first to the third quartiles. We see that for different thresholds, the MAD values of ZincSight are slightly smaller than those of Metal3D predictions.

**FIGURE 4:**
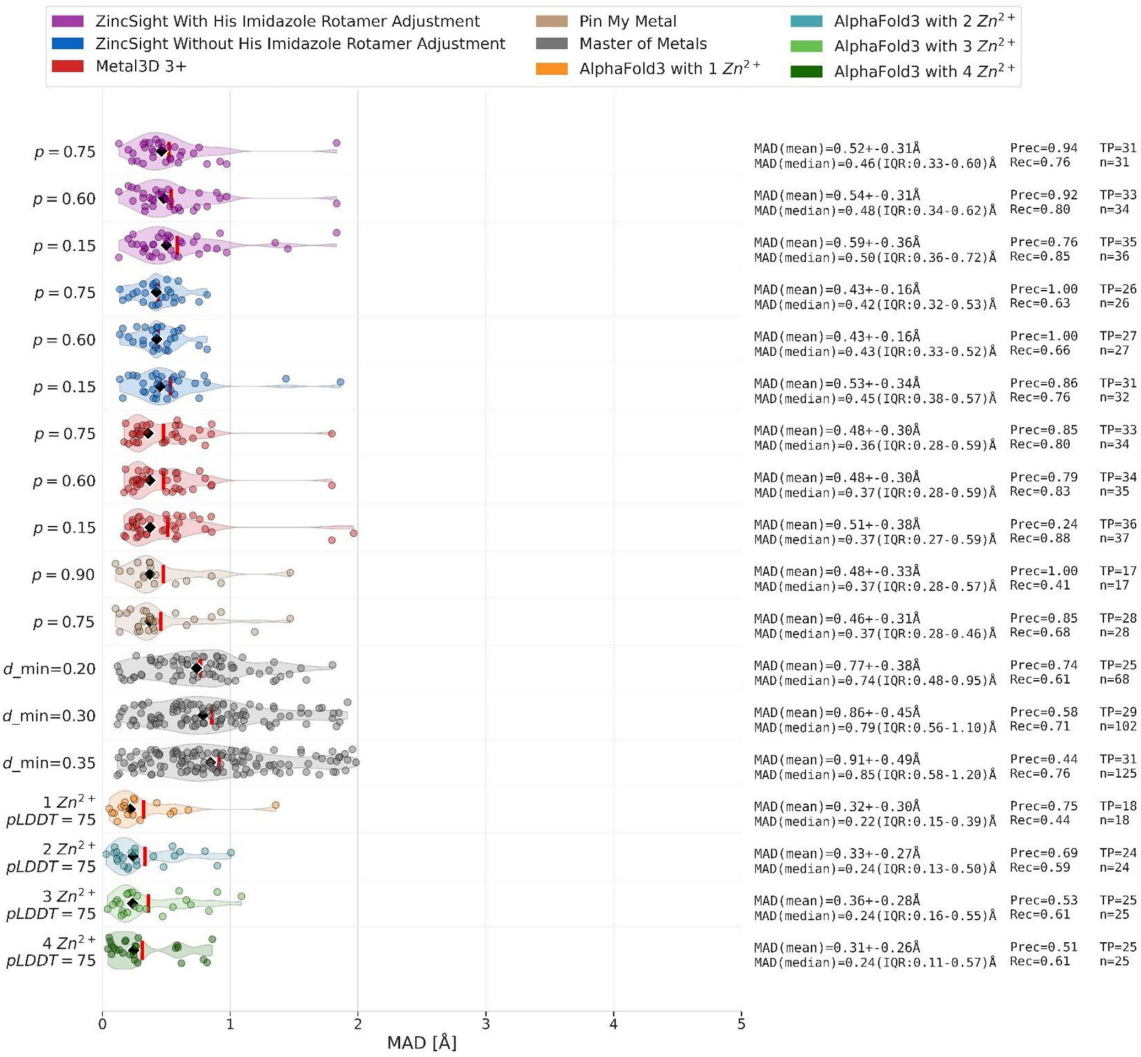
MAD for correctly predicted sites within 2 Å on test set 4. Calculated MAD for all zinc‐binding sites within the AF test set that ZincSight, Metal3d, MoM, PMM, and AF3 correctly predicted. MAD values were measured at probabilities estimates threshold of 0.75, 0.60, and 0.15 for ZincSight analyses with and without rotations. For Metal3D 3+ (red) with the same probability estimates thresholds, and for AF3 we considered a lower pLDDT threshold of 75 for each of the predicted zinc ions obtained by query with one to four zinc ions. Per each predicted probability threshold, n represents the number of the overall predicted metal sites within 2 Å of the actual zinc position. The number (*n*) of the actual predicted sites is represented by TP (multiple predicted sites can be within 2 Å radius from a single TP sites). Each dot represents the measured distance (in Å) between a predicted metal ion position and the actual zinc ion position. The violin shape shows the kernel density of these distances, with the red vertical line marking the mean and a black diamond shape marking the median.

We adopt and extend the evaluation protocol of Metal3D. Our analysis includes the PR curve (Figures 1, 2, Supplemental Figures S3, S4) and the mean absolute deviation (MAD mean) of distances between the predicted true positives (TP) and observed zinc ion positions (Figures 3 and 4, Supplemental Figures S5, S6). In our analysis, we also measured the median absolute deviation (MAD median), allowing comparison between measures sensitive to outliers (mean‐based) and more robust ones (median‐based).

We consider a prediction to be a TP if it is located within either a 2 or a 5 Å threshold of an experimentally verified zinc site, with multiple predictions for the same site counted as a single TP; the total number of TP predicted metal ions is marked by *n* in Figures 3 and 4 and Supplemental Figures S5, S6. If there is no predicted site within a 2 Å/5 Å radius of an experimentally determined site, it is categorized as a false negative (FN). Inaccurately positioned metals are marked as false positives (FP), yet clustered within a 2 Å/5 Å radius so that only one instance per cluster is counted. The 5 Å threshold follows the established Metal3D protocol. However, it encompasses a relatively large spatial volume, potentially causing predicted zinc ions to overlap with binding residues of other metal sites, which confuses the interpretation of multi‐nuclear zinc configurations. For example, statistical analyses between zinc ion distances in binuclear sites reveals a mean separation of 3.4–3.9 Å with standard deviation of 0.3–0.4 Å, suggesting that a 5 Å threshold might not differentiate well between multiple metal ion sites (Bazayeva et al., 2024; Yang et al., 2008). Rather, zinc ions typically exhibit coordination bond distances, generally ranging from 1.9 to 2.3 Å across various zinc‐ligand bonds (Laitaoja et al., 2013; Tamames et al., 2007). To better reflect these biologically relevant distances, we carried out a similar analysis using the stricter threshold of 2 Å for classifying predictions as TP. We evaluated the predictions with PR and MAD mean analyses using the 2 Å threshold (Figures 1, 2, 3, 4), and the 5 Å threshold (Supplemental Figures S3–S6).

### 2.4 Evaluating the zinc‐binding sites predictions, given an experimental structure (test set 1)

Figures 1 and S3 compare the PR curves of ZincSight and state‐of‐the‐art Metal3D using the 2 and 5 Å thresholds, respectively. The area under curve (AUC) of ZincSight is 0.86/0.90, with a precision of 0.85/0.90 at a recall of 0.85 when using the 2 Å/5 Å thresholds. For comparison, Metal3D has AUC of 0.80/0.83, and a precision of 0.68/0.77 at the same recall values, using the 2 Å/5 Å thresholds, yet exhibits higher precision in lower recall values, between 0.51 and 0.7.

Figures 3 and S5 show the MAD values of ZincSight's TP zinc predictions with respect to the experimentally determined positions. With a confidence threshold of *p* = 0.25, ZincSight detected 77 sites within a 2 Å radius of 77 verified sites, and predicted 80 zinc ion positions within a 5 Å radius of 79 verified sites, from a total of 86 experimentally verified sites. Location accuracy analysis showed that for *p* = 0.25 at 2 Å threshold predictions ZincSight had a MAD (mean) of 0.22±0.35 Å and MAD (median) of 0.11 Å (Interquartile range, IQR: 0.05–0.21 Å), while at the 5 Å threshold ZincSight had a MAD (mean) of 0.31±0.56 Å and MAD (median) of 0.12 Å (IQR: 0.06–0.23 Å), indicating a high spatial localization precision at both distance thresholds. For comparison, at the same confidence level of *p* = 0.25, Metal3D predicted 80 zinc ion positions within a 2 Å radius from 75 experimentally verified sites, resulting in a higher MAD (mean) of 0.47±0.42 Å and MAD (median) of 0.35 Å (IQR: 0.18–0.63 Å), while for the 5 Å threshold, it showed a MAD (mean) of 0.77±0.85 Å and MAD (median) of 0.39 (IQR: 0.20–0.79 Å) .

ZincSight is by far faster than Metal3D—the average prediction running time is 0.7 s per structure (with 250 residues on average) on a personal computer with 2 Intel i7 CPU cores. This is 36 times faster than the average 25 s that it takes Metal3D running on a multicore GPU workstation (20 CPUs, GTX2070) (Dürr et al., 2023). To our knowledge, ZincSight is currently the fastest structure‐based zinc ion binding site prediction tool.

### 2.5 Evaluating the selectivity toward non‐zinc ion metal species

The specificity of ZincSight zinc ion binding sites predictions compared to other metal‐ion binding sites has mixed outcomes. Test set 1 includes six PDB structures that bind both zinc and non‐zinc metal ions. In two of these structures, 4JJJ and 2ZJ6, ZincSight mistakenly predicted two experimentally characterized calcium‐ion binding sites (one in each structure) as zinc‐binding sites, with probabilities *p* = 0.30 (*s* = 15.32) and *p* = 0.37 (*s* = 14.75), respectively. Other non‐zinc metal‐ion binding sites within these two structures were not predicted. In the other four structures (4A7K, 5NOF, 5T77, and 5YZ4), none of the non‐zinc metal‐ion binding sites were confused with zinc‐binding sites.

**FIGURE 5:**
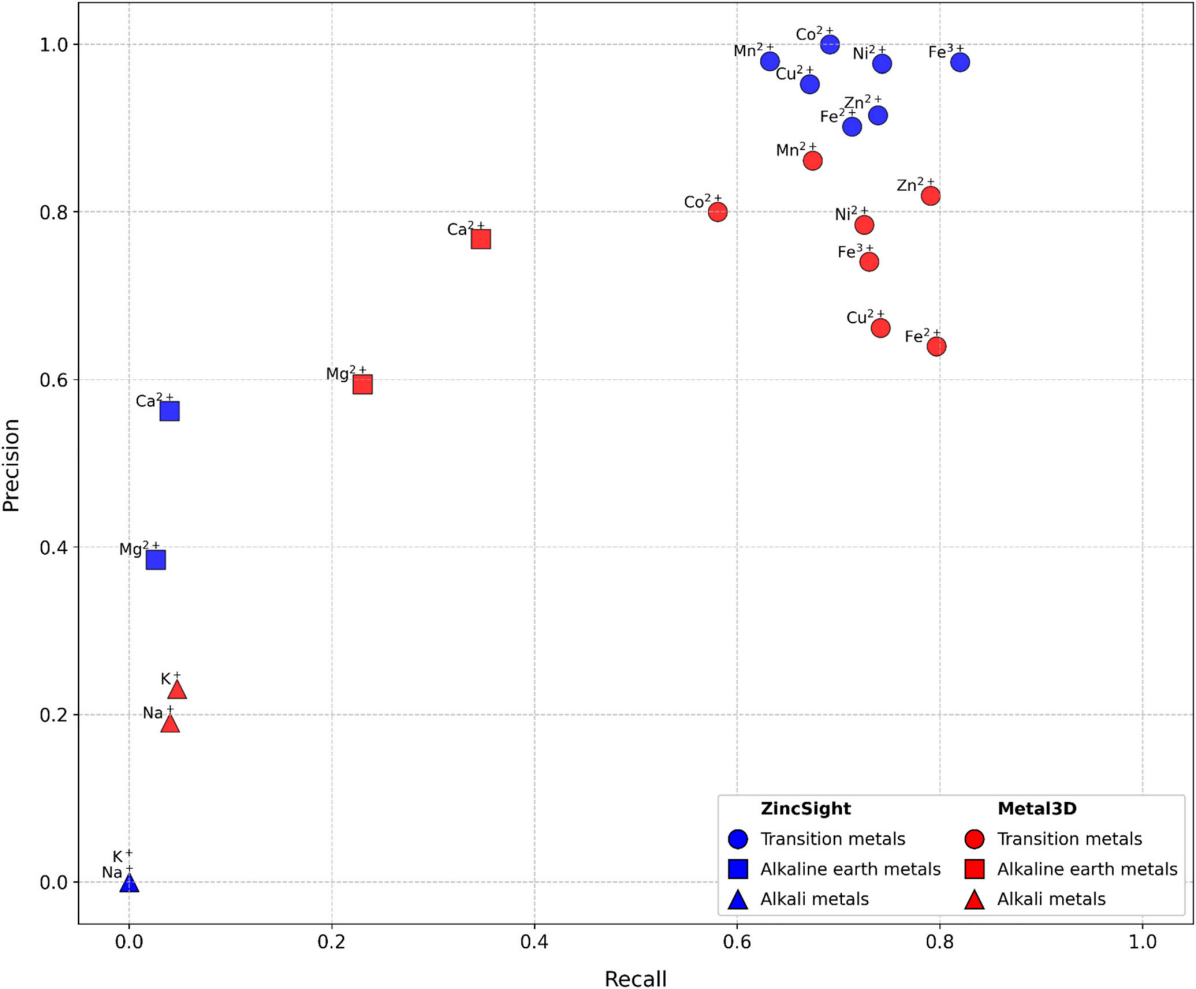
Metal‐ion species selectivity in test set 2. ZincSight selectivity toward other metal species, tested using the Metal3D test‐set and probability of *p* ≥ 0.75. ZincSight did not predict any alkali metal binding site. On the other hand, it predicted transition metals with high rates, and alkaline earth metals at low‐rate. ZincSight selectivity profile (blue) is somewhat improved compared to that of Metal3D (red).

To systematically evaluate ZincSight selectivity, we used test set 2, that is, the same panel of potential binding sites used for Metal3D, including alkali metals, alkaline earth metals, and a range of transition‐metal ions (Figure 5). At a probability threshold of *p* ≥ 0.75, ZincSight did not detect alkali‐metal coordination sites, as it should, and detected only some of the alkaline earth–metal sites. However, it consistently detected transition‐metal binding sites with high precision and recall values. Altogether, at this stringent probability threshold, ZincSight indistinguishably detected the binding sites of all transition metals, rather than just zinc.

### 2.6 Evaluating the sensitivity and specificity of ZincSight template library

We examined how well ZincSight's library of only 42 templates covers the broad spectrum of test set 3 binding sites in terms of sensitivity and specificity. Using a 0.5 probability threshold, ZincSight reached a sensitivity of 97.7%, correctly identifying 1551 of the 1588 true zinc‐binding sites, while generating 117 FP predictions. Of the 37 misses, 32 involved residue combinations covered by the 42‐templates library. However, their scores fell below the 0.5 threshold. ZincSight overlooked the other five binding sites because they featured amino acid combinations that were missing from the template library. In this respect, it is noteworthy that, to avoid relying on crystallographic artifacts, we opted to include in the 42‐template library only amino acid combinations that appear at least three times in the training set. This is beneficial for reducing the amount of FP predictions, possibly at the cost of FN predictions. The five sites that ZincSight overlooked comprise amino acid combinations that appeared less than 3 times in the training set: the combinations (“Asn,” “Thr,” “Thr”), (“Asn.” “Thr,” “Tyr”) and (“Glu,” “Glu,” “Ser”) that appeared only once, and the combination (“Asp,” “Asp,” “Tyr”) that appeared twice.

Of the 117 FP predictions, 77 (66%) correspond to other transition metals: 61 are copper‐binding sites and 16 are iron‐binding sites (Table S1). This further supports that ZincSight should best be thought of as a detector of transition metal binding sites in general rather than only zinc‐binding sites. One of the FP predictions corresponds to a calcium binding site. Interestingly, however, this calcium‐binding site, which was observed in the X‐ray crystal structure, is annotated in UniProt as a zinc‐binding site.

An additional 11 (9%) of the sites were classified as FP predictions because their zinc ion occupancy fell below 0.5 and were thus omitted from our reference ground‐truth dataset; these could be TP predictions that were misclassified. Two (1%) of the FP predictions were annotated in UniProt as zinc‐binding, but the zinc ion was not present in the X‐ray crystal structure. In 10 (9%) of the FP predictions, with three coordinating residues, only two actually bind zinc. These predictions are very closely related to true binding sites. The rest, 16, could be FP predictions. In summary, it appears that a significant portion of the 117 seemingly FP predictions, detailed in Supplemental Tables S1 and S2, may be true binding sites for zinc or other transition metals.

The analysis further demonstrated that ZincSight's 42‐template library, with its limitations, captures a diverse range of zinc‐binding modes, while reasonably balancing the FP and FN predictions. This is consistent with large‐scale data mining of metal‐ion environments in proteins, which demonstrated that a relatively small set of coordination patterns accounts for the vast majority of biologically relevant sites (Bowman et al., 2016; Zhang & Zheng, 2020). In light of these observations and to further ensure future adaptability, in local execution, ZincSight enables users to add their tailor‐made metal‐binding templates to the library when predicting binding sites.

### 2.7 Prediction of zinc‐binding sites in test set 4 of AF models

Figure 2 shows the PR curves for ZincSight predictions on the AF2 test set with and without rotamer optimization. For comparison, we also evaluated Metal3D, MoM, and PinMyMetal (PMM) predictions for the same AF2 test set models. Also, we queried AlphaFold 3 (AF3) with the protein sequences of this test set, running four separate queries per protein (the queries vary by the number of zinc ions specified between one and four). At both the 2 and 5 Å thresholds, ZincSight with rotamer optimization yielded the greatest gains in high‐recall regions. Specifically, at 2 Å (Figure 2), precision at 0.8 recall rised from 0.80 without adjustment to 0.92 with it, while at 5 Å (Figure S4) it increased from 0.82 to 0.97 (Table 1). Overall, rotamer adjustment slightly improved the AUC‐PR at 2 Å (0.85 vs. 0.83; Table 1), and led to a more substantial gain at 5 Å (0.98 vs. 0.90); two examples for the improved performance are shown in Figure S7. Incorporating histidine rotamer optimization comes with a modest increase in FP predictions between 0.6 and 0.8 recall (Figure 2). However, the clear advantage in high‐recall regions, makes it a compelling choice.

**TABLE 1:** PR metrics for detecting zinc‐binding sites within 2 Å in test set 4 (AF2 models).

Method	2 Å			5 Å		
	Precision at 0.8 recall	Recall at 0.85 precision	AUC	Precision at 0.8 recall	Recall at 0.85 precision	AUC
**ZincSight with rotation**	**0.92**	**0.81**	**0.85**	**0.97**	**0.95**	**0.98**
**ZincSight without rotation**	**0.80**	**0.76**	**0.83**	**0.82**	**0.80**	**0.90**
Metal3D 3+	0.92	0.83	0.82	0.92	0.86	0.93
Master of metals	0.00	0.56	0.68	0.98	0.86	0.85
PinMyMetal	0.00	0.68	0.68	0.00	0.76	0.75
AlphaFold 3 (1 Zn)	0.00	0.02	0.39	0.92	0.83	0.77
AlphaFold 3 (2 Zn)	0.00	0.37	0.58	0.64	0.56	0.76
AlphaFold 3 (3 Zn)	0.00	0.49	0.59	0.46	0.59	0.72
AlphaFold 3 (4 Zn)	0.34	0.51	0.64	0.38	0.56	0.69

*Note*: PR values are shown for ZincSight (with and without histidine‐imidazole rotamer optimization; bold fonts), Metal3D, MoM, PMM and AF3. Ground‐truth zinc coordinates were obtained by superimposing experimentally determined zinc‐binding sites from the corresponding PDB structures (matched by UniProt accession) onto the AF2 models.

Among the FN predictions, that is, actual zinc‐binding sites that ZincSight failed to predict, were the proteins Q7MVV4 and Q9NXF7. In both cases, AF2 assigned low pLDDT confidence to the coordinating residues, reflecting reduced structural model accuracy that likely diminished ZincSight's sensitivity for detecting these binding sites. More details are found in the “Effect of model accuracy on performance” section below.

Figure 4 shows the MAD values for the TP predictions by the different methods for test set 4 (AF2 models). Generally speaking, the different methods placed their true predictions in accurate positions, characterized by low MAD values. The most accurate was AF3 with exceptional spatial accuracy‐ MAD (median) ~0.22–0.25 Å (Figures 4 and S6), albeit as seen in Figure 2, with significantly lower recall (0.44–0.66). Metal3D was second in spatial accuracy at probability of 0.75 with MAD (median) of 0.36 (Figures 4 and S6), with higher recall (0.80–0.83). PMM ranked third in spatial accuracy, with spatial accuracy in probability of 0.75 with MAD (median) of 0.37 (Figures 4 and S6), with lower recall (0.68–0.76). ZincSight (with and without rotamer adjustment) ranks fourth in accuracy, achieved MAD values around 0.5 Å. MoM exhibited poorer localization − MAD (median) 0.74–1.28 Å at the examined recall range of 0.61–0.85 (Figures 4 and S6).

Collectively, these results demonstrate that at both 2 Å (Figure 2) and 5 Å (Figure S4) thresholds, ZincSight with rotamer adjustment demonstrates a modest precision advantage over Metal3D. On the other hand, Metal3D produced slightly lower MAD values than ZincSight, both in terms of the median and mean values (Figures 4 and S6).

In test set 4, the average protein length is 485 amino acids, and the average ZincSight running time per structure was 0.87 s with histidine imidazole rotamer adjustment and 0.47 s without it. For MoM, the average run‐time per structure was 2.56 s; PMM required 17.4 s, and Metal3D was slowest with an average of 39.8 s per structure.

#### 2.7.1 Effect of model accuracy on performance

We ran ZincSight with 753 non‐redundant protein models (test set 5), selected from UniRef50 clusters, that were released after the AF2 monomer training cutoff date of April 30, 2018. We categorized binding site predictions into high‐confidence (mean pLDDT ≥80) and low‐confidence (mean pLDDT <80) classes, and then quantified TP, FP, and FN predictions in each. For FN counts, we averaged the pLDDT over the AF residues corresponding to PDB‐validated zinc ion sites. At a probability threshold of 0.5, the FP:TP ratio was essentially independent of predicted confidence: 860 TP versus 56 FP for high‐confidence sites, and 10 TP versus 1 FP for low‐confidence sites, whereas the TP:FN ratio declined in the low‐confidence group (860 TP vs. 296 FN at ≥80; 10 TP vs. 20 FN at <80) (Figure S8).

#### 2.7.2 A comment regarding AF3's prediction of zinc ion binding sites

AF3 is a diffusion‐based architecture for protein structure prediction from sequence (Abramson et al., 2024). Unlike AF2, its predecessor, AF3 can also predict structures of protein complexes and the small molecules bound to them, including metal ions, and zinc in particular. A key limitation of AF3's ligand‐binding site prediction is its requirement for user‐specified protein‐ligand stoichiometry. This feature can complicate zinc‐binding site predictions when the number of binding sites is unknown, necessitating multiple prediction runs for a single structure. Below we show three examples that we encountered while using AF3 on our test sets and where we made the realistic assumption that we do not know the number of zinc‐binding sites. These examples demonstrate that AF3's zinc‐binding site predictions heavily depend on the user‐specified stoichiometry.

- The protein Q86T03 has two verified zinc‐binding sites (PDB structure 8OQH, residues 72–159). We predicted its structure with AF3 and specified one to four zinc ions. Querying the server with this protein and two zinc ions resulted in correct prediction of the two zinc ion binding sites, both assigned with high pLDDT confidence scores: 97.03 and 97.82. A query with a single zinc ion also resulted in locating the zinc ion correctly, with a pLDDT of 77.49. However, when queried with four zinc ions, AF3 predicted two sites, correctly, with high pLDDT scores of 98.32 and 98.59, and two additional incorrect sites assigned with pLDDT of 77.05 (comparable with the confidence assigned in the case of the single zinc) and 43.29. Hence, the number of specified ligands can influence prediction confidence scores. In comparison, ZincSight correctly identified both true sites as highly likely to bind zinc, assigning them with probabilities of *p* = 0.89 (*s* = 9.81) and *p* = 0.90 (*s* = 9.61).
- Protein Q8NBJ9 has one verified zinc‐binding site (PDB structure 7Y63). A query with one zinc ion correctly identified the site, assigning it with a score of 90.66, but this dropped to 82.19 when four zinc ions were listed in the query. This highlights the stoichiometry dependency of AF3 and the need for multiple queries for accurate results. In comparison, ZincSight, successfully predicted only the true zinc‐binding site assigning it with a probability of *p* = 0.79 (*s* = 11.27).
- Protein Q08281 (PDB structure 8ADL) presents an interesting case where AF3 assigned consistently low pLDDT scores across all predicted zinc‐binding sites, regardless of the number of zinc ions in the query. Despite containing three verified zinc‐binding sites, even queries specifying the correct stoichiometry produced poor predictions, with the highest pLDDT of 33.68 for a true binding site. Queries with two zinc ions yielded even lower scores, while four‐ion queries showed only marginal improvement, reaching a maximum pLDDT of 35.41. In contrast, ZincSight correctly identified all three binding sites, with probabilities of *p* = 0.99 (*s* = 4.12), *p* = 0.94 (*s* = 8.52), and *p* = 0.91 (*s* = 9.33).

#### 2.7.3 USING ZincSight

A Colab notebook (https://colab.research.google.com/github/MECHTI1/ZincSight/blob/master/ZincSight.ipynb) offers interactive and streamlined access to ZincSight. Colab's limitations on runtime and computational resources may render it unsuitable for processing very large input queries, such as a whole proteome. In such scenarios, the software is available on GitHub for download (https://github.com/MECHTI1/ZincSight) and can be run locally. The local mode supports adding entries to the template library. Users can provide structure files (in mmCIF or PDB format) or list the structure IDs to be automatically downloaded from the PDB, AFDB, or ESMFold DB. The default setting of ZincSight that explores rotameric states per histidine to enhance sensitivity can be toggled off.

The execution output is an archive file with PyMOL sessions and a CSV format summary table. The PyMOL sessions show the predicted zinc ions as colored spheres within the input structure. The color scheme of the spheres reflects their confidence score (probability) for being true zinc‐binding sites, transitioning from red (low confidence) through yellow to green (high confidence). The summary table also lists the template with a similar zinc‐binding site that the predictions are based upon to support the results. Users can initially interpret the results using the table and use the PyMOL sessions for visualization.

#### 2.7.4 Example 1—Using ZincSight Colab for prediction on AF “dark cluster” sample

We analyzed protein structures representing “dark clusters” identified by Barrio‐Hernandez et al. (Barrio‐Hernandez et al., 2023). In their comprehensive study of the AFDB, they clustered 214 million proteins into 2.3 million structural groups and identified 711,705 clusters (31%) as “dark clusters” that show no significant structural similarity to known PDB structures and lack matches to Pfam domains or existing annotations in UniProt/TrEMBL and SwissProt databases. Of these clusters, 1057 are of size 70 or more, that is, including more than 70 similar proteins. We analyzed one representative in each of these clusters.

**FIGURE 6:**
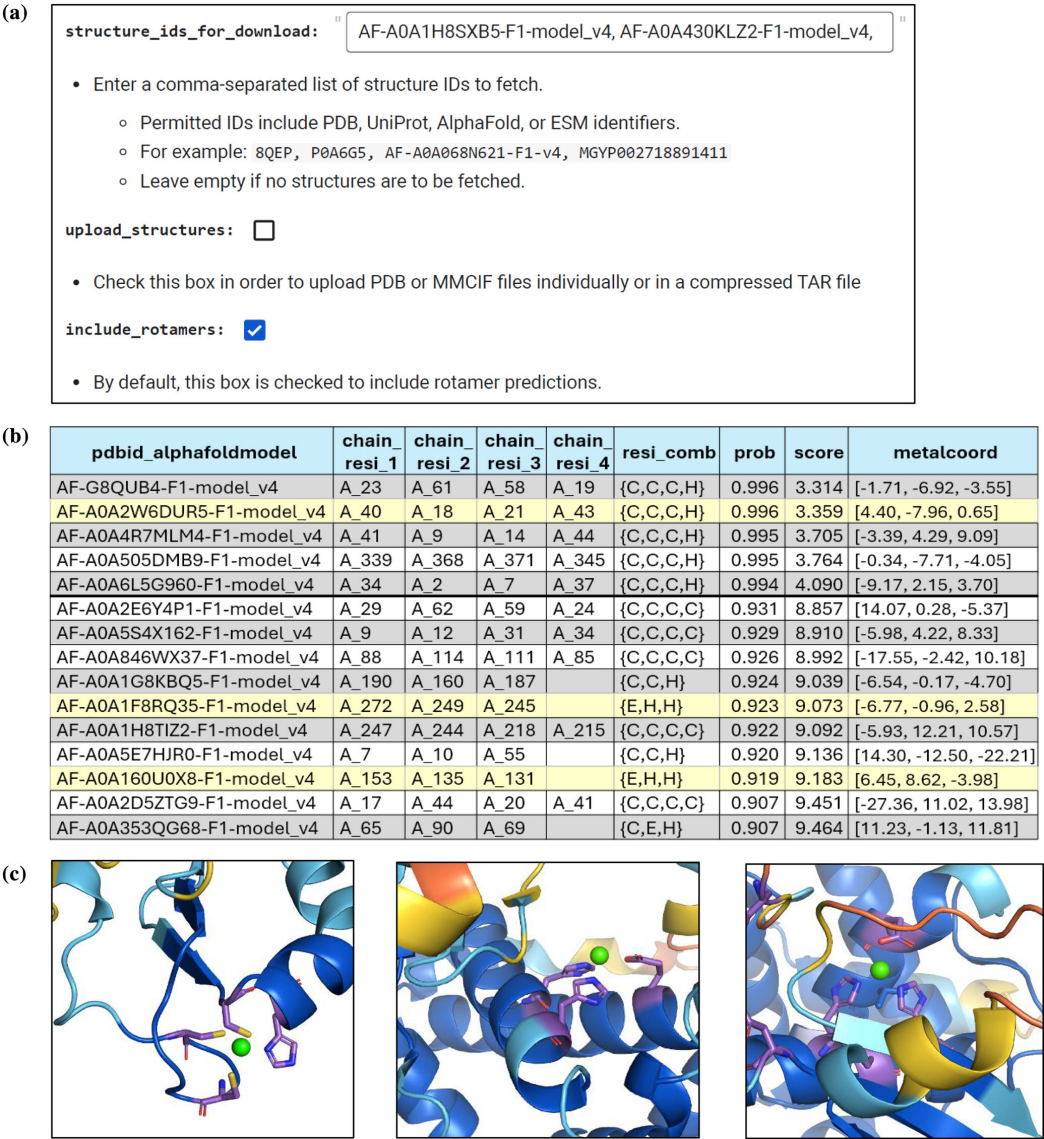
The ZincSight pipeline using the Google Colab interface and its resulting output. (a) The input interface provided within the Google Colab environment. (b) Prediction results displayed as a table. (c) Predicted zinc ion binding sites within the AF2 structural models AF‐A0A2W6DUR5‐F1‐model_v4 (left), AF‐A0A1F8RQ35‐F1‐model_v4 (middle) and AF‐A0A160U0X8‐F1‐model_v4 (right). All were assigned high likelihoods to be real: p=0.996 (s=3.36), p=0.92 (s=9.07), and p=0.9187 (s=9.18), respectively.

To run the predictions for all 1057 UniProt accession IDs via Colab, we entered all IDs (Figure 6) and selected the “include_rotamers” option. The software installation in the Colab environment took 1 min and 28 s, downloading the structural input models from the AF database required 1 min and 32 s, and the ZincSight predictions completed in 10 min and 28 s.

Figure 6c illustrates the zinc‐binding site predictions for the proteins AF‐A0A2W6DUR5‐F1‐model_v4, A0A160U0X8, and A0A1F8RQ35. Both A0A2W6DUR5 and A0A160U0X8 are assigned as “uncharacterized proteins” without any annotations indicating zinc ion or metal‐binding activity. On the other hand, A0A1F8RQ35 is identified as a “DUF885 domain‐containing protein” through Google ProtNLM (Gane et al., 2022). This Pfam classification associates it with the Peptidase_MA clan, which is composed of zinc‐dependent metallopeptidases characterized by an HExxH motif.

The predicted zinc‐binding sites in both A0A1F8RQ35 and A0A160U0X8 contain three residues typically associated with catalytic zinc ion binding (Daniel & Farrell, 2014), specifically, two histidine residues and one glutamate residue. The two histidine residues are part of the HExxH motif (x being any residue), commonly linked to metallopeptidases (Cerdà‐Costa & Xavier Gomis‐Rüth, 2014). While this observation aligns well with the Pfam classification of A0A1F8RQ35 in the Peptidase_MA clan, A0A160U0X8 lacks Pfam annotation or any functional description of enzymatic activity. We used Metal Activity Heuristic Of Metal and Enzyme Sites (MAHOMES II) (Feehan et al., 2023) to assess the potential catalytic function of A0A160U0X8. MAHOMES II is a structure‐based machine learning framework that predicts whether metal‐binding sites are enzymatic or non‐enzymatic. It uses a gradient boosting classifier trained on structural features and incorporates 10 different models with varied random seeds to enhance prediction robustness. For each site, MAHOMES II calculates the percentage of models classifying the site as enzymatic and provides an overall determination. The zinc ion binding sites predicted by ZincSight for A0A1F8RQ35, A0A160U0X8, and A0A2W6DUR5 structural models were submitted to MAHOMES II. The A0A1F8RQ35 and A0A160U0X8 models were classified as enzymatic with 100% consensus across all prediction models, indicating high catalytic potential. In contrast, the zinc‐binding site in A0A2W6DUR5 was classified as non‐enzymatic by all prediction models.

All the zinc‐binding sites were assigned a very high probability of being correct. However, given the lack of prior evidence that the proteins bind metal ions or exhibit enzymatic activity, laboratory‐based experimental validation is needed to confirm these computational predictions.

#### 2.7.5 Example 2—metagenomic driven proteins sampling

To evaluate ZincSight's ability to discover previously unannotated zinc‐binding sites in metagenomic proteins, we randomly selected 200 MGnify‐only clusters of AFESM, a clustered database of all predicted structures from the AF and ESM‐Fold databases (Yeo et al., 2025). We purposefully selected only clusters lacking AF entries. ZincSight predicted 12 sites at a threshold of *p* ≥ 0.75, and 19 sites at a threshold of *p* ≥ 0.50 (Table S3), corresponding to 11 and 16 unique proteins, respectively. This is a manifestation of the usefulness of ZincSight for analyzing metagenomic data. Obviously, the predicted binding sites should be viewed as hypotheses requiring further examination.

For benchmarking, we also conducted an mebipred (*p* ≥ 0.50) search on the same dataset, identifying 11 hits. Four of these hits, MGYP001130174518, MGYP001351529692, MGYP002623597247, and MGYP002639704763, emerged also in our ZincSight search, assigned with high probabilities of *p* ≥0.75.

One may wonder whether the rest of the 7 hits that emerged in the mebipred search are true binding sites, or perhaps false positives. Providing a full answer is obviously beyond the scope here, but to partially address it, we used AF3 to model the structures of two of the proteins (MGYP001381295889 and MGYP000243407481) in complex with zinc ions. In both predicted co‐complexes, zinc binding is coordinated by two residues only: a Glu‐Glu pair in one instance, and a Glu‐Cys pair in the other. These two putative binding modes depart from the canonical tetrahedral geometry, suggesting that both are more likely transient contacts than bona fide zinc‐binding sites.

## 3 DISCUSSION

We introduced ZincSight, a software tool for analyzing protein structures in search of putative binding sites for zinc and other transition metals. ZincSight can predict transition metal locations in model structures and was validated with respect to experimentally determined structures. ZincSight is available for download or on Google Colab. It is comparable to alternatives in sensitivity and accuracy, as indicated by the PR on the test set and zinc ion location accuracy metrics, while running significantly faster. For example, ZincSight processed batches of experimentally solved structures, of 485 residues on average speed of 0.87 and 0.47 s per protein, with and without histidine‐rotamer exploration, respectively, on a personal computer using 2 Intel i7 CPU cores. Thus, it is suitable for proteome‐wide analysis of transition metal‐binding sites. Such large‐scale application is valuable for characterizing unannotated proteins, where the presence of metal‐binding sites can provide functional insight.

ZincSight was designed based on the hypothesis that zinc binding is local and determined purely based on stereochemistry. Thus, the known zinc‐binding sites were clustered based on their amino acid composition and stereochemistry, resulting in 42 templates. The detection of a cluster that is similar enough to one of these templates is taken as an indication of zinc binding. The success of this approach validates our hypothesis. A second (implicit) hypothesis is that the currently documented zinc‐binding sites are inclusive, in the sense that these 42 templates balance the FP and FN predictions. Trusted newly emerging forms of zinc binding will be added as additional templates, and users can proactively add templates they trust to supplement their own searches. Of note, ZincSight output includes both the predicted sites and the template zinc‐binding sites that they are based on. Currently, ZincSight applies rotamer analysis only to histidine residues, due to their high prevalence in zinc‐binding sites. The application of rotamer analysis to other coordinating residues could improve prediction accuracy further. Examination of the degree to which the backbone and side chains of a template overlap with the predicted site provides a direct means of interpretation. This is a meaningful advantage of ZincSight with respect to its machine‐learning‐based alternatives, which do not provide any such interpretability.

Zinc binding might be mediated by multiple subunits, each of which contributes one or more metal coordination bonds. To be able to detect such cases, the full oligomeric structure should be provided as input. This is relevant for homo‐oligomers, where zinc binds at the interface between subunits. Analyzing the monomeric structure might not suffice because the partial zinc‐binding site that remains would be assigned a low probability to be considered real. For example, the Rad50 protein has been shown to form a homodimer via common zinc ion binding sites between its monomers, with each monomer contributing two Cys residues to bind the zinc ion (Hopfner et al., 2002). Similarly, in *Medicago truncatula* L‐histidinol dehydrogenase, the zinc‐binding site spans the interface of two monomers: two residues, a histidine and an aspartate, come from one monomer, while another histidine comes from the other (Ruszkowski & Dauter, 2017). A ZincSight query using the monomeric AF2 models of these two proteins (P58301 and G7IKX3) did not yield any predicted zinc ion binding site. Thus, in cases where the zinc ion binding motif is shared by more than one chain, the multimeric form of the protein should be used as the query. Inherently, ZincSight analysis of predicted structural models is challenging since some residues within the binding sites may be misoriented.

Previous work has established that although some characteristics are unique to specific metal ion binding sites, many such sites share common structural features (Cerdà‐Costa & Xavier Gomis‐Rüth, 2014; Dudev & Lim, 2014; Laveglia et al., 2023). This is particularly evident for transition metals, where binding site promiscuity is often observed due to similarities in coordinating residue side‐chain conformations. Such overlap in binding capabilities among different metal ions presents a significant challenge for predictive tools aiming to achieve high selectivity, like ZincSight. Indeed, ZincSight detects the location of zinc and all other transition metals with similar accuracy. Thus, it can best be thought of as a means to detect binding sites of any transition metal, rather than just zinc. In this respect, it is noteworthy that metal selectivity in proteins is influenced not only by binding site geometry but also by external factors, such as environmental metal ion concentration (Bromberg et al., 2022; Dudev & Lim, 2014; Ruszkowski & Dauter, 2017; Waldron and Robinson, 2009), interactions with metallochaperones (Dudev & Lim, 2014; Laveglia et al., 2023; Ruszkowski & Dauter, 2017; Waldron and Robinson, 2009; Weiss et al., 2022), and subtle variations in the secondary coordination shell (Barwinska‐Sendra et al., 2020; Dudev & Lim, 2014; Feehan et al., 2023; Sendra et al., 2023).

External tools, such as AF‐Multimer (Homma et al., 2023) and the more recent AF3, make it straightforward to generate multimeric models for zinc‐binding analysis. AF3 can also predict zinc‐protein co‐complexes; however, the zinc:protein stoichiometry has to be specified. ZincSight analysis, on the other hand, is not based on stoichiometry. Thus, preliminary ZincSight analysis may guide AF3 in that it could quickly outline candidate zinc‐binding proteins, as well as suggest the stoichiometry. Our examination shows that, provided with the correct stoichiometry, AF3's prediction is more accurate (Figure 4). In this respect, ZincSight is complementary to AF3.

ZincSight was designed to facilitate the analysis of batches of protein queries in the search for zinc‐binding sites. One of our immediate goals is to extend its application to the systematic detection of zinc‐binding sites in uncharacterized proteins by leveraging advanced protein models generated by AF (for which we showed it fares well) or ESMFold. Indeed, the AFDB and the ESM Metagenomic Atlas (Lin et al., 2023) include hundreds of millions of predicted protein structures, readily available for ZincSight analysis. With that, ZincSight can uncover novel Zn^2+^ coordination arrangements, including multi‐nuclear arrangements, thereby providing valuable templates for artificial metalloprotein design. Finally, ZincSight can be used to test various hypotheses. For example, it may enable insights and discoveries both in evolutionary gain and loss of zinc‐binding sites among protein families and organisms.

## 4 METHODS

### 4.1 Identification of candidate zinc‐binding sites

In proteins, zinc binding requires a specific chemical coordination, typically in a tetrahedral arrangement, though it can occasionally exhibit 5‐ or 6‐coordination geometry (Laitaoja et al., 2013). Zinc binding is mostly mediated by the side chains of histidine (His), cysteine (Cys), aspartate (Asp), and/or glutamate (Glu) residues. More specifically, zinc ions are predominantly coordinated by the sulfur atom of Cys, the nitrogen atom of His, or the carboxylate anions of Asp and Glu. Other amino acids, such as asparagine (Asn), glutamine (Gln), threonine (Thr), and tyrosine (Tyr), though less common in zinc‐binding sites, tend to bind zinc via their polar uncharged side chains (Dudev & Lim, 2014; Laitaoja et al., 2013). ZincSight uses an inverted index structural motif search method inspired by Bittrich et al. (2020). Our inverted index has only the first two query features of the four used by Bittrich et al.: residue types and distances between their respective C*α* and C*β* atoms. As the inverted index strategy significantly reduces search space, querying is very fast.

Given a query structure, ZincSight identifies and collects all potential zinc‐binding residues by selecting pairs that satisfy the following two conditions: (1) their type is known to mediate zinc ion binding (His, Glu, Cys, Asp, Thr, Tyr, Ser, or Asn (Laitaoja et al., 2013); Supplemental Table S4), and (2) the distance between the potential zinc‐binding atoms of their side chains is lower than 7 Å. For residues with two typical potential zinc‐binding atoms, the average location of these atoms is used (e.g., OE1 and OE2 for Glu). ZincSight then searches for these collected potential zinc‐binding sites in the inverted index with the values from our curated 42 zinc‐binding structural templates. This lax binding site definition reduces the complexity and speeds up the search, and potentially uncovers more zinc‐binding sites.

Approximately one‐third of the zinc ions present in crystal structures are estimated to be artifacts (Laitaoja et al., 2013; Laveglia et al., 2023). These artifacts are non‐biological byproducts introduced during experimental processes where, zinc ions from crystallization buffers or precipitants bind to protein surfaces, often with incomplete coordination spheres. Such binding primarily results from zinc ions facilitating crystal formation and packing by stabilizing intermolecular contacts or reducing conformational flexibility. Thus, for our templates set, we considered cases that had multiple similar instances of zinc‐binding sites in the training set, and selected an instance among them to be included in the template set at random.

The templates were extracted from the Metal3D training set, which contains X‐ray crystal structures with resolution better than 2.5 Å. While B‐factors were initially considered as a filtration criterion, they were ultimately excluded from our selection process. This decision was made because zinc ions are sometimes located within dynamic structures, such as enzymatic active sites, where B‐factors may be relatively high despite the biological relevance of these sites. Including such sites in both training and test sets was deemed important for comprehensive coverage of zinc‐binding environments.

### 4.2 Zinc ion localization

#### 4.2.1 Pipeline for prediction of zinc ion positions

ZincSight uses the template zinc‐binding site stored in the inverted index to predict the location of the zinc ion in the query structure as follows:

- First, we calculate an initial estimate of zinc ion location. For zinc‐sites with four or more residues, the starting point is the calculated arithmetic mean of the binding atoms. For residues with more than one candidate atom, the average location of the relevant atoms is used. For sites with three binding residues, we use two starting points one on each side of the plane formed by the three binding atoms and along the normal vector passing through the calculated arithmetic mean. Here, we select two starting points because these two symmetric points have identical distances to the atoms defining the plane. For candidate sites with three residues that include a histidine residue, ZincSight determines the favorable zinc ion location based on the spatial configuration of the imidazole group(s). In (the relatively rare) cases where a candidate site with three residues does not include a histidine residue, that is, in 8 of the 42 templates, one of the two possible zinc ion locations is arbitrarily selected. (The next version of ZincSight will use both locations.)
- For each binding residue, we consider the atom that binds zinc. For residues with two typical zinc‐binding atoms, we select the atom nearest to the above estimated location of the zinc ion as the zinc‐binding atom.
- We refine the position of the zinc ion based on the optimal distances for its coordination. We use 2.15 Å as the ideal coordination distance for atoms in all amino acids, except the S atom of cysteine, where we use 2.32 Å. These distances are within one standard deviation from the mean of the real distances in natural zinc‐binding environments (Laitaoja et al., 2013; Tamames et al., 2007; Yao S et al., 2015). To place the zinc ion with minimal deviation from these predefined distances, we use the non‐linear least squares optimization with the Levenberg–Marquardt method (Levenberg, 1944) and minimize $\sum_{i=1}^{n}{\left(d_{i}-d_{\text{ideal}}\right)}^{2}$, where n is the number of measured distances between the to‐be placed zinc ion and its candidate ligands. In this function, $d_{i}$ is an individual coordination bond length, and $d_{\text{ideal}}$ is the ideal distance. Finally, ZincSight includes an option of sampling histidine imidazole group rotamers to identify favorable rotamer configurations, as described in Section 4.3.1.2.

#### 4.2.2 Evaluating the training‐set error

We evaluated the accuracy of ZincSight predictions on the training set (excluding the 42 template structures). This validation is part of our training procedure and is akin to validation set error. Supp. Figure S9 shows the MAD values of the distances between predicted zinc ion locations in correctly predicted sites, within the training set, to the experimentally measured positions. We also verified that the distributions of distances between the zinc ion and its binding atoms, as well as the angles formed between zinc ions and histidine imidazole groups, are similar in the training set experimental data and the ZincSight predictions (Supplemental Figures S10 and S11).

### 4.3 Scoring and probability estimation for candidate zinc‐binding sites

The candidate zinc placements are scored according to two or three features: (1) the distances between the candidate binding atoms to the predicted zinc ion location, (2) the structural similarity between a candidate zinc‐binding site to an experimentally solved structural template, with the same zinc‐binding residue combination, and (3) for candidate sites that contain histidine residue(s), spatial orientation of the histidine imidazole group(s) relative to the predicted location of the zinc ion.

#### 4.3.1 Score features

##### Feature 1: distances between predicted zinc ion to candidate binding ligands

The RMSD of the differences between the prediction distances ($d_{i}$) in the *n* candidate sites to the pre‐determined “Ideal Distance Value” ($d_{\text{ideal}}$), listed above $d_{\text{RMSD}}=\sqrt{\frac{1}{n}\sum_{i=1}^{n}{\left(d_{i}-d_{\text{ideal}}\right)}^{2}}$.

##### Feature 2: structural similarity to the zinc ion binding site templates

For each candidate binding site, we use Biopython's *PDB.QCPSuperimposer package* (Cock et al., 2009) to optimally superimpose the zinc‐binding atoms of the candidate position and the template and calculate the RMSD between them, denoted $T_{\text{RMSD}}$.

##### Feature 3: histidine angles score

**FIGURE 7:**
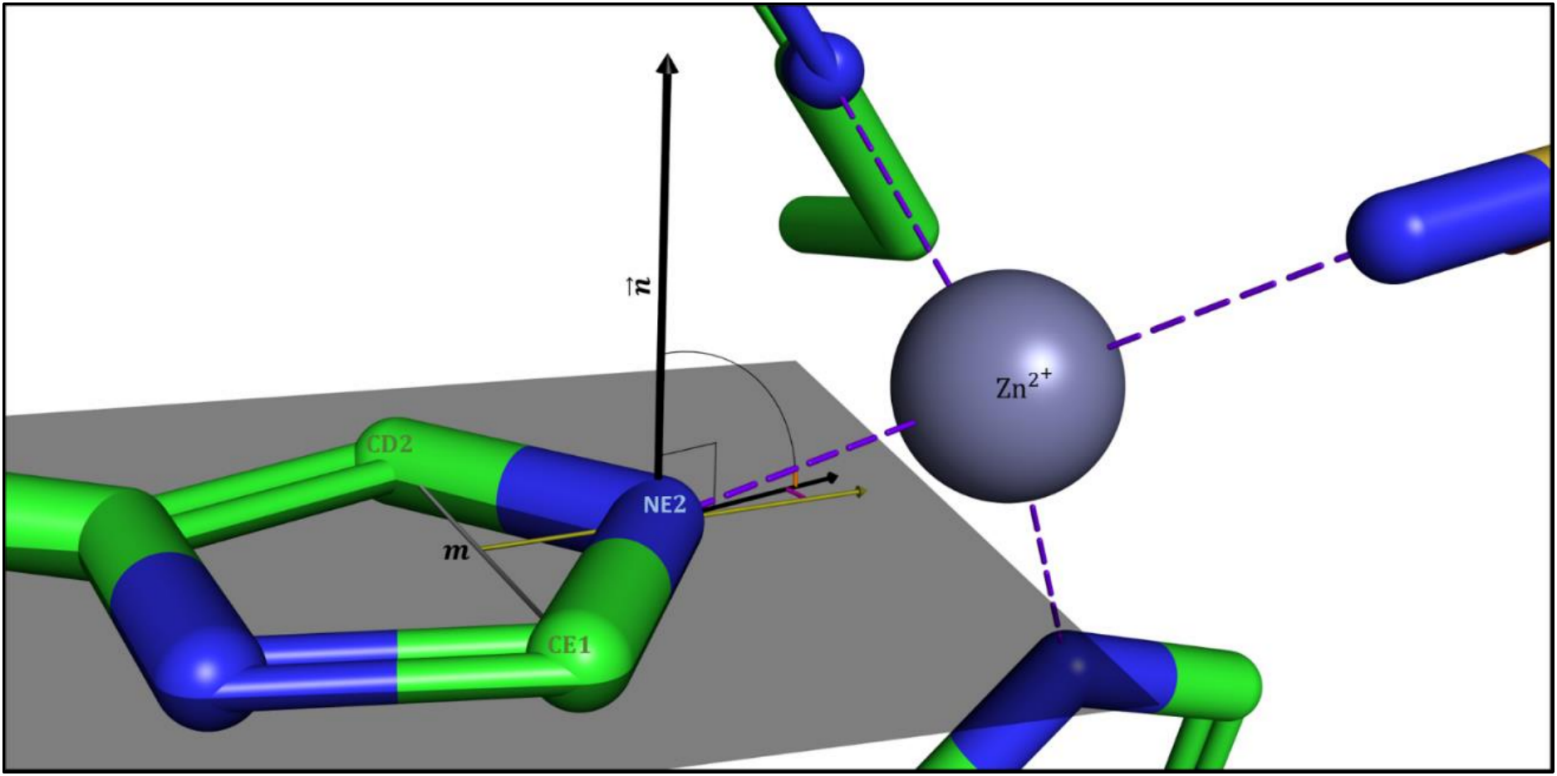
The angles between the histidine imidazole and zinc. Depiction of the angles between the coordination bond vector and the imidazole side chain of histidine. “Angle to Plane,” marked as *α* (orange): Angle between the theoretical coordination bond to the histidine's imidazole side chain plane (gray). This angle is calculated as 90° minus the angle between the imidazole plane normal ($\vec{n}$) and the theoretical coordination bond vector. “Angle to $\vec{\mathrm{mN}}$,” marked as *β* (magenta): The angle between extended vector of $\vec{\mathrm{mN}}$ (yellow) to the projection of the predicted coordination bond vector on the imidazole plane.

The spatial orientation of the histidine imidazole is measured by two angles:

- The angle between the predicted coordination bond's vector (formed between the positioned zinc ion to the candidate binding atom) and the histidine side chain imidazole group(s). It is denoted “Angle to Plane” and marked *α* in Figure 7.
- The angle between the projection of the predicted coordination bond vector on the imidazole plane to the vector crossing the midpoint between carbons “C1” and “C2” and the predicted zinc‐binding nitrogen “N” (defined as the extended vector of $\vec{\mathrm{mN}}$, where *m* denotes the midpoint between the two carbon (C) atoms). When N = NE2, C1 is CD2 and C2 is CE1, and when N = ND1 C1 is CE1 and C2 is CG. It is denoted “Angle to $\vec{\mathrm{mN}}$” and marked β in Figure 7.
  We use RMS to combine the differences between the *n* computed angles and the pre‐calculated ideal angle values (from established data (Chakrabarti, 1990)) into a single value (Equations (2), (3)). RMS is more suitable than the mean absolute error (MAE) for our data (Jakeš, 1988; Theobald, 2005) because the errors follow a normal distribution (Chai & Draxler, 2014), calling for a greater penalty for large errors. The angle scores are:

(1) $$\text{Angles to Planes}\;{\left(\alpha \right)}_{\mathrm{RMS}}=\sqrt{\frac{1}{n}*\sum_{i=1}^{n}{\left(\text{Angle}\;\text{to}\;\text{Plane}\;{\left(\alpha \right)}_{i}\right)}^{2}\;}$$

(2) $$\text{Angles to}\;\vec{\mathrm{mN}}\;{\left(\beta \right)}_{\mathrm{RMS}}=\sqrt{\frac{1}{n}*\sum_{i=1}^{n}{\left(\text{Angle to}\;\vec{\mathrm{mN}}{\left(\beta \right)}_{i}\right)}^{2}}$$

And these RMS values are then summed:

(3) $$\mathrm{His}\;\text{Angles Score}=\begin{array}{l} \text{Angles to Planes}\;{\left(\alpha \right)}_{\mathrm{RMS}}\\ +\;\text{Angles to}\;\vec{\mathrm{mN}}{\left(\beta \right)}_{\mathrm{RMS}} \end{array}$$

and used to select the better, that is, lower score, location between the two possible positions in candidate sites with three residues.

#### 4.3.2 Adjustments to predicted AF structure models by sampling histidine rotamers

To improve the prediction of zinc‐binding sites for structure models predicted by AF2 that have a histidine residue, ZincSight samples rotameric states. Sampling hypothetical rotamers can compensate for inaccurate predictions of rotameric states. To this end, we examine the imidazole group's rotational space around the CG–CB axis (χ2) by 180° degrees to find rotamer configurations that minimize deviation from ideal “His Imidazole Angles.” Then, ZincSight iteratively samples these configurations in 15° increments, selecting the optimal configuration at each step based on predefined ideal His Imidazole Angles. To avoid re‐examining previously evaluated configurations, each iteration focuses exclusively on new rotational configurations, omitting those evaluated in prior iterations. We have accelerated the procedure by multithreading, enabling parallel execution of multiple sampling processes across two cores.

Note that this rotamer sampling process does not consider statistical distributions of the histidine dihedral angles (χ1, χ2, etc.) (Chakrabarti, 1990; Li & Hong, 2011; Shapovalov & Dunbrack, 2011). We exclude these factors because rotamer libraries include data from both apo (unbound) and holo (bound) protein structures, which do not account for the zinc ion's effect on the binding residues' conformations. Additionally, structural models, including those predicted by AF2 may be inaccurate, including in their backbone conformation, for proteins that exist in both apo and holo forms (Saldaño et al., 2022). Given that the rotamer sampling is initialized from a potentially inaccurate initial histidine conformation, employing a broad spectrum of rotamer configurations can facilitate the identification of imidazole conformations that support zinc binding, thereby overcoming inaccuracies in the initial residue conformation.

#### 4.3.3 Calculating zinc‐binding sites scores

##### Definition of the predictive model formulas

ZincSight score s of a candidate zinc‐binding site combines the feature scores with the parametrized weights *b*1, *b*2, and *b*3, and a constant *c*:

(4) $$s=\begin{array}{l} b1\times \left(d_{\text{RMSD}}\right)+b2\times \left(T_{\text{RMSD}}\right)+b3\\ \times \left(\mathrm{His}\;\text{Angles Score}\right)+c \end{array}$$

The values of these parameters depend on whether the zinc ion binds three or at least four residues. For each case the values of *b*1, *b*2, *b*3, and *c* were optimized separately. If the candidate zinc‐binding site does not contain a histidine residue, we weigh the Histidine Angle Score component with *b3* = 0. Table 2 lists the parameters we use in these four distinct cases.

**TABLE 2:** Optimized parameters for zinc‐binding site prediction models.

Equation #	Include histidine	3 or 4+ residues	b1	b2	b3	c
1	✓	4+ residues	12.3	5.0	0.3	0
2	✓	3 residues	13.8	0.7	0.2	8.1
3	✕	4+ residues	13.8	14.5	0	5.5
4	✕	3 residues	12.9	1.6	0	14.4

*Note*: This table provides the optimized parameters (*b1*, *b2* , *b3* , and *c* for four predictive models used to compute the ZincSight score for zinc ion binding sites. The models are tailored based on whether histidine residues are included and whether the binding site consists of three residues or four or more residues, with parameters calibrated using Optuna's TPE to maximize the PR‐AUC on a validation dataset.

##### Hyperparameter optimization

The parameters were calculated to optimize the AUC for the PR curve on a validation set of 2043 structures (structures in the Metal3D training set that are not among the 42 structures that contributed the representative structural templates). In these evaluations, we considered a TP prediction as one that included all binding residues of the actual binding site. Sites that were only partially predicted, that is, where only some of the coordination residues were correctly predicted, were excluded from the count. Such sites were not included in the TP or FP counts. For example, a prediction that included three residues within an actual four‐residue zinc‐binding site was neither a TP nor a FP. Counting such matches as TP could lower the model's efficiency in accurately identifying and scoring predictions with complete matches against partial ones. Alternatively, categorizing these near matches as FP might compromise the model's effectiveness in detecting genuine sites that consist of exactly three residues, due to the close resemblance between the configurations of partially matched and actual three‐residue sites.

We used the TPE algorithm (Bergstra J et al., 2011) for hyperparameter optimization, implemented in Optuna (Akiba et al., 2019). We applied Optuna's TPE algorithm (“TPEsampler”). We specified the resulted PR‐AUC value of the validation set as the maximization objective. We set the algorithm to perform 10,000 subsequent trials. The calibration process, coupled with an exploration of the parameters space, was conducted to identify their optimal values. The search covered a range from 0 to 15, with increments of 0.1, for both coefficients and the constants. The resulted coefficients and constants which yield the maximal AUC value have been selected.

#### 4.3.4 Transforming zinc‐binding sites scores into probabilities

##### Calibration of Platt scaling model

We use Platt scaling to convert the raw prediction scores, for potential zinc‐binding sites, into probabilities (Platt et al., 1999). Platt scaling is a widely used probability scaling method (Lin et al., 2007) that transforms raw scores into posterior probabilities by fitting a parametric logistic regression model, without requiring explicit density estimation.

We selected Platt scaling for its simplicity and effectiveness (Böken, 2021). In our implementation, we mapped the classifier raw scores onto predicted probabilities that represent the likelihood of a site to bind zinc. The posterior probability $p_{i}$ of *i*th sample with score $s_{i}$ being classified in the positive class (y=1), that is, a zinc‐binding site is modeled as:

(5) $$p_{i}=P\left(y=1|s_{i}\right)=\frac{1}{1+e^{-\left(As_{i}+B\right)}}$$

The scaling parameters, A and B, are determined by minimizing the negative log‐likelihood (NLL), equivalently maximizing the log‐likelihood, over the training set $\left\{\left(s_{i},y_{i}\right)\right\}$, with $y_{i}\in \left\{0,1\right\}$. This objective NLL function measures how well the scaled probabilities explain the observed outcomes:

(6) $$\underset{A,B}{\text{argmin}}\;\left\{\sum_{i=1}^{N}\;\;\left[y_{i}\log\left(p_{i}\right)+\left(1-y_{i}\right)\log\left(1-p_{i}\right)\right]\right\}$$

Conceptually, this function comprises two main penalties:

1. $y_{i}\log\left(p_{i}\right)$: Penalizes low probabilities (*p*_*i*_≈0) for true zinc‐binding sites (*y*_*i*_=1).
2. $\left(1-y_{i}\right)\log\left(1-p_{i}\right)$: Penalizes high probabilities (*p*_*i*_≈1) for negatives (non‐zinc‐binding sites, $y_{i}$ = 0).
3. N is the total number of samples in the dataset.
  We performed the scaling process on the same dataset used for the predictive scoring model for the hyperparameter optimization. We used a random split of the dataset into training (80%) and validation (20%) subsets. The training set was used to fit the Platt scaling model, while the validation set was reserved for evaluating calibration performance. The calibration curve and the resulted probability distribution illustrate that the resulted model effectively achieves a probability estimation for our method (Supplemental Figure S1).

##### Evaluation of the calibrated model

We evaluated the success of the Platt scaling calibration process, by the Brier Score and LogLoss metrices. $\text{Brier Score}\;\left(\mathrm{BS}\right)$ (Brier, 1950) measures the MSE between predicted probabilities and observed outcomes:

(7) $$\mathrm{BS}=\frac{1}{N}\sum_{i=1}^{N}{\left(f_{i}-o_{i}\right)}^{2}$$

where:

1. $f_{i}$ is the calibrated probability for the *i*th sample.
2. $o_{i}$ is the observed label (0 or 1).
3. N is the total number of samples in the dataset.

This metric ranges from 0 (perfect calibration) to 1 (maximum error). In our work, the Brier Score was 0.0346, indicating that, on average, the predicted probabilities closely tracked the true outcomes. A Brier Score below 0.05 is typically considered excellent in binary classification.

LogLoss (Niculescu‐Mizil & Caruana, 2005) penalizes heavily predictions that are both confident and wrong. It is calculated as:

(8) $$\mathrm{Log}\;\text{Loss}=-\frac{1}{N}\sum_{i=1}^{N}\left[o_{i}\ln\left(f_{i}\right)+\left(1-o_{i}\right)\ln\left(1-f_{i}\right)\right]$$

Lower values indicate better calibration, with 0 representing a perfectly predicted probability distribution. Our model's LogLoss of 0.1163 suggests that the predicted probabilities are highly reliable. This result demonstrates that the calibration procedure successfully mitigated overconfident, incorrect predictions.

#### 4.3.5 *Threshold selection to maximize F1 and F*β *scores*

To select threshold scores that will serve as a benchmark for identifying zinc‐binding sites, we looked for prediction scores where the F1 and F*β* (*β* = 2) scores reach their maximum. The $F1=2\times \frac{\text{precision}\times \text{recall}\;}{\text{precision}+\text{recall}}$ score, defined as the harmonic mean of precision and recall, provides a balanced measure of the model's performance (Lever J et al., 2016). The $\mathrm{F\beta}=\left(1+\beta^{2}\right)\times \frac{\text{precision}\times \text{recall}}{\left(\beta^{2}\times \text{precision}\right)+\text{recall}}$ score generalizes this concept by introducing a parameter *β* that adjusts the weight of recall relative to precision. These scores were derived from PR curves evaluating the algorithm's performance in successfully identifying at least three binding residues, as well as in predicting zinc ions within a range of 5 Å of the actual (Supplemental Figures S12a and S13). For tasks where precision and recall are equally important, we recommend using predictions that yield the maximum F1 for both performance parameters matrices (0.88–0.91), corresponding to a prediction threshold in the range 0.39 ≤ *p* ≤ 0.41. Conversely, for tasks where recall is prioritized over precision—aiming to minimize missed zinc‐binding occurrences—we suggest using the predictions that achieve the maximum F*β* (*β* = 2) (0.86–0.89), specifically those with *p* ≤ 0.20 (Supplemental Figures S12a and S13). This ensures that fewer potential binding sites are overlooked, although it may allow more false positives.

Compressing zinc ion binding site predictions: selection of favorably scored sites within a defined radius cluster

To minimize overlaps in the predicted binding sites, such as a three‐residue site within a four‐residue site, we employed a selection strategy for predicted zinc ions. We prioritize zinc ions that achieved the best score relative to others within a 2.6 Å radius. A radius of 2.6 Å was selected based on published data, which indicates that over 99% of the distances between zinc ions in binuclear sites exceed 2.6 Å (Yang et al., 2008). We select the zinc ions iteratively until only one zinc ion was present within a 2.6 Å radius sphere, thus enhancing the accuracy of the predictions and avoiding redundancy in identifying binding sites.

### 4.4 Runtime complexity

ZincSight's runtime complexity can be analyzed by examining each step individually. Index construction cost is $O\left(L\right),$ since ZincSight performs one pass over the L residues to build an inverted index that assign each residue once for each pair combination of amino acids. Each residue is represented by the number of inverted indexes with residues within our selected threshold radius. Candidate retrieval, which depends on the total number of index hits (F), is bounded as $O\left(L\right)$ due to a constant upper limit (M). On pattern matches, if there are histidine residues, histidine imidazole group rotamers exploration is executed, such that, in each round spawns up to $3h_{i}$ new χ‐angle states (exploring 0°, ±15° per histidine). This refinement step is performed iteratively until the cumulative rotational adjustment approaches 180°. This refinement complexity scales exponentially with the number of histidine residues ($h_{i}$) per motif, but since this is bounded ($h_{i}$ ≤4), refinement remains a constant factor $O\left(3^{h_{\max}}\right)$ such that:

$$\begin{array}{rcl} O\left(L\right)+O\left(F\right)+i & = & \sum_{1}^{F}O\left(3^{h_{i}}\right)\\ & \leq & O\left(L+\left(M\cdot L\right)\cdot 3^{h_{\max}}\right)=O\left(L\cdot 3^{h_{\max}}\right) \end{array}$$

The term $3^{h_{\max}}$ is constant, and the runtime scales linearly with the protein length (L). This theoretical linear scaling is confirmed by runtime measurements (Figure S14). A regression analysis of computational runtimes for batches of AF2‐predicted protein models clearly demonstrates this linear relationship, yielding a strong coefficient of determination of $R^{2}=0.971$. The experimental data thus validates the predicted linear scaling of ZincSight's runtime with increasing protein length. We used a batch of 300 AF model structures with average size of 500 residues to test memory usage. The maximal memory for the batch was 192 Megabyte, both with, and without, the histidine rotamers exploration step.

### 4.5 Constructing test set 4 of AF2 models

Starting with 1783 UniProt accessions containing zinc‐bound protein structures released after April 30, 2018, we chose the longest protein from each UniRef50 cluster, resulting in 753 proteins. We then grouped these 753 proteins by distinct Pfam families that did not have a structure released before the same date. For a Pfam family that met the criterion, we first prioritized those that were reviewed in UniProt (Swiss‐Prot). If no Swiss‐Prot‐reviewed protein was available, we selected the longest chain as the representative; if several were Swiss‐Prot reviewed, we chose the longest. Importantly, we chose AF models that corresponded to PDB structures not belonging to any UniRef50 cluster or Pfam family with members released prior to the end of both AF Monomer v2.0's and ZincSight's training sets timelines (i.e., April 2018 and March 2021, respectively). One model (ID: Q96JC1) was excluded due to its corresponding PDB structure (ID: 6ZE9) not being solved in its native form. These structural models were generated using AF Monomer v2.0, and are available in the AF database and listed in Supplemental Table S5.

To assess the accuracy of predicted zinc‐binding sites, we conducted structural superimpositions between the zinc‐binding regions of the 27 experimentally determined PDB structures and their corresponding AF models. For each PDB structure, we identified segments composed of seven‐residue windows centered on the zinc‐binding residues, extending three residues in both directions. These segments were superimposed onto their corresponding segments in the AF models, including the bound zinc ion. The position of the zinc ion in the AF structure served as a reference point for evaluating the accuracy of the predicted zinc ion location.

## AUTHOR CONTRIBUTIONS

**Gilad Mechtinger:** Software; writing – original draft; writing – review and editing; conceptualization; methodology; data curation; validation; visualization; formal analysis; investigation. **Gabriel Axel:** Writing – original draft; software; writing – review and editing; conceptualization; methodology; data curation; validation; investigation; formal analysis. **Rachel Kolodny:** Writing – original draft; writing – review and editing; conceptualization; methodology; data curation; validation; investigation; formal analysis. **Nir Ben‐Tal:** Writing – original draft; project administration; supervision; resources; writing – review and editing; conceptualization; methodology; validation; formal analysis; investigation; funding acquisition; data curation.

## Supporting information


**Table S1:** Breakdown of the 117 false‐positive predictions by ZincSight (*p* ≥ 0.5) on the coverage evaluation set of 830 x‐ray crystal structures (≤ 2.5 Å; 1588 true Zn^2+^ sites). Categories: copper‐binding, iron‐binding, calcium site annotated as zinc‐binding site, low‐occupancy zinc‐binding sites (occupancy < 0.5), predictions with only two actual ligation residues, UniProt‐annotated zinc‐binding sites lacking zinc ion in the structure, and other cases.
**Table S3:** ZincSight predictions (*p* > 0.5) for 200 randomly selected representatives of the AFESM “only ESM” clusters.
**Table S4:** A list of the amino acids and specific atoms known to mediate zinc ion coordination (Bowman et al., 2016; Dudev & Lim, 2014). ZincSight considers these residues and the specified atoms.
**Table S5:** Test set 4; the AlphaFold2 test set of protein structures used for evaluating zinc‐binding site predictions. The table provides AlphaFold ID, PDB ID, UniProt ID, and Pfam ID for each protein.
**Figure S2:** Distribution of the RMSD values of the predicted zinc‐binding sites of the AF2 test set (test set 4) relative to the corresponding true PDB‐solved sites. The analysis targeted specific regions of the PDB structures encompassing zinc‐binding residues and their adjacent three‐residue sequence neighbors, superimposing these onto corresponding regions of the AF2 models. The mean RMSD was 0.60 Å, indicating a high degree of structural similarity. The variance of 0.64 Å is skewed by two sites with considerably high RMSD values (~4 Å). These higher RMSD values correspond to AF2 structures where parts of their zinc‐binding residues are misoriented relative to the superimposed PDB structure. Despite this, the vast majority of RMSD values are below 1 Å. This structural similarity highlights the accuracy of AF2 models, particularly within zinc‐binding site regions, enabling ZincSight to reliably predict most zinc‐binding sites in this set.
**Figure S3:** PR in identifying zinc‐binding sites within 5 Å in test set 1. PR for ZincSight (blue curve) and Metal3D (red curve) predictions for all 86 “3+ zinc sites” of the test set of 59 proteins structures. The probability thresholds p used for predictions are marked. ZincSight maintains high precision across different recall values. Metal3D has a somewhat higher precision in recall region ~0.5‐through‐~0.75, and ZincSight has higher precision above recall = ~0.75.
**Figure S4:** PR in identifying zinc‐binding sites within 5 Å in test set 4 (AF2 models). The PR curves for ZincSight, Metal3D, MoM, PMM, and AF3 prediction tasks for zinc‐binding sites within this test set. PR metrics for ZincSight predictions were computed for the prediction task with (purple curve) and without (blue curve) optimization of the rotameric state of the histidine imidazole. The ground truth zinc ions coordinates are determined by superimposing zinc‐binding sites from each model's corresponding PDB structure, sharing the same UniProt accession, onto the AF2 model. Looking at the full spectrum, ZincSight is on par with, perhaps slightly better then, the best alternatives.
**Figure S5:** MAD for correctly predicted sites within 5 Å in test set 1. Calculated MAD values for all 86 “3+ zinc sites” in the Metal3D test set that were correctly predicted by ZincSight (blue) and Metal3D (red). ZincSight's MAD values measured at set of predicted probabilities thresholds ranging from 0.75 to 0.15. Per each probability threshold, *n* is the number of the overall predicted metal sites within 5 Å of the actual zinc position. TP is the number of correctly predicted sites (multiple predicted sites can be within 5 Å radius from a single TP site). Each dot represents the measured distance (in Å) between a predicted metal ion position and the actual zinc ion position. The violin shape shows the kernel density of these distances, with the red vertical line marking the mean and a black diamond shape marking the median. We see that for different thresholds, the MAD values of ZincSight are smaller than those of Metal3D predictions.
**Figure S6:** MAD for correctly predicted sites within 5 Å on test set 4 (the AF models). Calculated MAD for all zinc‐binding sites within the AF test set that ZincSight, Metal3d, MoM, PMM and AF3 correctly predicted. MAD values were measured at probabilities estimates threshold of 0.75, 0.60, and 0.15 for ZincSight analyses with and without rotations. For Metal3D 3+ (red) we used the same probability estimates thresholds. For AF3 we considered a pLDDT threshold of 75 for each of the predicted zinc ions obtained by query with stoichiometry of one‐through‐four zinc ions per protein. For each predicted probability threshold, *n* represents the number of the overall predicted metal sites within 5 Å of the actual zinc position. The number of the actual predicted sites is represented by TP (multiple predicted sites can be within 5 Å radius from a single TP sites). Each dot represents the measured distance (in Å) between a predicted metal ion position and the actual zinc ion position. The violin shape shows the kernel density of these distances, with the red vertical line marking the mean and a black diamond shape marking the median.
**Figure S7:** Comparison of zinc ion predictions in example AF models with and without His rotamer sampling. As an example of predictions in AF models, we show the AF2 prediction (purple) of *Homo sapiens* SIDT2 protein (AF ID: AF‐Q8NBJ9‐F1) (a, b) and *Vibrio cholerae* ZrgA protein (c, d) (AF ID: AF‐Q9KP27‐F1), superimposed with their corresponding experimental PDB structures (cyan) (IDs: 7Y63 and 8F1B, respectively). Predictions are shown with (a, c) and without (b, d) rotamer sampling. Experimentally determined zinc ions (dark‐gray spheres) from PDB structures are shown for reference. Predicted zinc ion locations are colored according to their ZincSight‐assigned predicted probabilities. Probability colorbar is provided, ranging from red (unfavorable, low probability), through yellow, to green (favorable, high probability). Implementation of rotamer sampling led to more favorable scores for zinc ion binding sites in both SIDT2 (green sphere, a) and ZrgA (yellow sphere, c) compared to predictions without rotamer sampling (orange and red spheres in b and d, respectively). In this example, ZincSight demonstrates its ability to predict zinc ion locations even when AF models' histidine imidazole group is initially misoriented.
**Figure S8:** Correlation between AF2 mean pLDDT and structural similarity of predicted zinc‐binding sites to their experimental counterparts. Scatter plot of mean pLDDT versus RMSD between PDB‐validated zinc‐coordinating residues and their AF2‐predicted counterparts for each site (test set 5). Points are colored by outcome: TP (green) have the predicted zinc ion within 2 Å of the experimental ion upon superposition; FP (orange) lack any PDB zinc within 2 Å; FN (blue) are experimental sites with no prediction within 2 Å. Zinc‐binding sites located in regions with mean pLDDT below 80 display lower structural accuracy. ZincSight is much more likely to overlook these compared to sites in regions assigned pLDDT above 80.
**Figure S9:** Distance between predicted and actual zinc ion location within the training set. MAD of distances between predicted zinc ion locations in correctly predicted binding sites, within the training set, to actual zinc ion location. Only sites for which all zinc‐binding residues have been correctly predicted, have been taken into account in this analysis. The small MAD values attest to the accuracy of ZincSight in placing the zinc ions near their real positions. The violin shape shows the kernel density of the distances, with the red vertical line marking the mean and a black diamond shape marking the median.
**Figure S10:** Distributions of two types of angles (in degrees) between predicted/resolved zinc ions and histidine imidazole groups within predicted and resolved zinc‐binding sites. The histograms illustrate two key angles: Angle_to_Plane (*α*) (a, b) and Angle_to_$\vec{\mathrm{mN}}$ (*β*) (c, d), used for guiding zinc ion placement through the incorporation of ideal constraints on coordination bond angles, based on established data (Zhang & Zheng, 2020). These angles, defined in Section 3.1.3 and depicted in Figure 7, were measured across zinc‐binding sites in the training set. Angle_to_Plane (*α*) is the angle between the coordination bond vector and the imidazole plane, while Angle_to_$\vec{\mathrm{mN}}$ (β) represents the angle between the coordination bond vector and the vector from the midpoint of imidazole carbons to the nitrogen atom coordinating the zinc ion. Two distributions are analyzed for each angle: (i) Panels (a) and (c), show the distributions for correctly predicted zinc‐binding sites, encompassing all binding residues and their predicted zinc ion positions. (ii) Panels (b) and (d) correspond to resolved zinc‐binding sites from PDB structures. The high similarity between the predicted and resolved distributions, validats the use of ideal angle constraints for zinc ion positioning, and demonstrates ZincSight's accuracy in reproducing experimentally determined preferences for coordination bond angles within zinc‐binding sites.
**Figure S11:** Distribution of zinc‐ligand distances (Å) in predicted and resolved zinc‐binding sites. The histograms present the distributions of distances (Å) between predicted/resolved zinc ions and their binding ligands within both predicted (a) and resolved (b) zinc‐binding sites in the training set. Predicted sites are those that include all actual binding residues. ZincSight utilizes specified optimal zinc‐ligand coordination bond lengths to position zinc ions within candidate sites. These distances are based on available data (Bowman et al., 2016; Ye et al., 2022). The majority of measured zinc‐ligand distances fall within 2.0 to 2.5 Å for both predicted and resolved sites. The similarity consistency validates ZincSight's zinc ion positioning approach and confirms the suitability of the selected ideal zinc‐ligand.
**Figure S12:** Identification of zinc‐binding sites' residues in the training set. PR curve for ZincSight prediction task for all zinc‐binding sites within 2043 protein structures in the training set. (a) TP predictions are defined as predicted binding sites where at least three predicted binding residues match the experimentally verified binding residues, and all predicted residues are involved in zinc‐binding. FP predictions are defined as cases where fewer than three of the predicted binding residues are involved in zinc‐binding, or when not all predicted binding residues at the same site are actually involved in binding. For example, a prediction with four binding residues where only three of the predicted residues bind zinc. (b) TP predictions are defined as predicted binding sites where all predicted binding residues match the experimentally verified binding residues. FP predictions are defined as cases where not all experimentally verified binding residues are included within the prediction, or when not all predicted binding residues are involved in zinc‐binding. Maximal F1 and F*β* (*β* = 2) scores, along with their corresponding ZincSight scores, are annotated for both curves.
**Figure S13:** Identification of zinc‐binding sites within 5 Å in the training set. Precision‐Recall curve for ZincSight prediction task for all “3+ zinc‐binding sites” within 2043 protein structures within the training set. TP predictions are defined as predicted sites within 5 Å of an experimentally verified zinc site. FP predictions within a 5 Å radius were clustered and counted as a single instance per cluster. Maximal F1 and F*β* (*β* = 2) scores, along with their corresponding ZincSight scores, are annotated on the curve.
**Figure S14:** Run‐time scaling with protein size (residue number). A scatter plot depicting computational run times for batches of 300 proteins of varying sequence lengths. The evaluated structures are AF2‐predicted protein models, randomly selected from UniProt queries based on length categories indicated by labeled ticks, each encompassing a range of ±1 residues around the specified length. A linear regression fit to the data yields a coefficient of determination ($R^{2}$) of 0.971, demonstrating that run‐time scales linearly with protein length within the tested range.

**Table S2:** Residue‐level details of the 117 false‐positive predictions summarized in Table S1, listing the predicted zinc‐binding residues for each case.

## Data Availability

The data that support the findings of this study are openly available in MECHTI1/ZincSight at https://github.com/MECHTI1/ZincSight.

## References

[pro70350-bib-0001] Abramson J , Adler J , Dunger J , Evans R , Green T , Pritzel A , et al. Accurate structure prediction of biomolecular interactions with AlphaFold 3. Nature. 2024;630(8016):493–500. 10.1038/s41586-024-07487-w 38718835 PMC11168924

[pro70350-bib-0002] Akiba T , Sano S , Yanase T , Ohta T , Koyama M . Optuna: A next‐generation hyperparameter optimization framework. Proceedings of the 25th ACM SIGKDD international conference on knowledge discovery & data mining. Anchorage, AK, USA: ACM; 2019. p. 2623–2631. 10.1145/3292500.3330701

[pro70350-bib-0003] Andreini C , Banci L , Bertini I , Rosato A . Counting the zinc‐proteins encoded in the human genome. J Proteome Res. 2006;5(1):196–201. 10.1021/pr050361j 16396512

[pro70350-bib-0004] Aptekmann AA , Buongiorno J , Giovannelli D , Glamoclija M , Ferreiro DU , Bromberg Y . mebipred: identifying metal‐binding potential in protein sequence. Bioinformatics. 2022;38(14):3532–3540. 10.1093/bioinformatics/btac358 35639953 PMC9272798

[pro70350-bib-0005] Barrio‐Hernandez I , Yeo J , Jänes J , Mirdita M , Gilchrist CLM , Wein T , et al. Clustering predicted structures at the scale of the known protein universe. Nature. 2023;622(7983):637–645. 10.1038/s41586-023-06510-w 37704730 PMC10584675

[pro70350-bib-0006] Barwinska‐Sendra A , Garcia YM , Sendra KM , Baslé A , Mackenzie ES , Tarrant E , et al. An evolutionary path to altered cofactor specificity in a metalloenzyme. Nat Commun. 2020;11(1):2738. 10.1038/s41467-020-16478-0 32483131 PMC7264356

[pro70350-bib-0007] Bazayeva M , Andreini C , Rosato A . A database overview of metal‐coordination distances in metalloproteins. Acta Crystallogr D Struct Biol. 2024;80(5):362–376. 10.1107/S2059798324003152 38682667 PMC11066882

[pro70350-bib-0069] Bergstra J, Bardenet R , Bengio Y , Kégl B . Algorithms for hyper‐parameter optimization. Proceedings of the 25th International Conference on neural information processing systems. Granada, Spain: Curran Associates Inc; 2011. p. 2546–2554.

[pro70350-bib-0008] Berman HM . The Protein Data Bank. Nucleic Acids Res. 2000;28(1):235–242. 10.1093/nar/28.1.235 10592235 PMC102472

[pro70350-bib-0009] Bittrich S , Burley SK , Rose AS . Real‐time structural motif searching in proteins using an inverted index strategy. PLoS Comput Biol. 2020;16(12):e1008502. 10.1371/journal.pcbi.1008502 33284792 PMC7746303

[pro70350-bib-0010] Böken B . On the appropriateness of Platt scaling in classifier calibration. Inf Syst. 2021;95:101641. 10.1016/j.is.2020.101641

[pro70350-bib-0011] Bowman SEJ , Bridwell‐Rabb J , Drennan CL . Metalloprotein crystallography: more than a structure. Acc Chem Res. 2016;49(4):695–702. 10.1021/acs.accounts.5b00538 26975689 PMC4838947

[pro70350-bib-0012] Brier GW . Verification of forecasts expressed in terms of probability. Mon Wea Rev. 1950;78(1):1–3. 10.1175/1520-0493(1950)078<0001:VOFEIT>2.0.CO;2

[pro70350-bib-0013] Bromberg Y , Aptekmann AA , Mahlich Y , Cook L , Senn S , Miller M , et al. Quantifying structural relationships of metal‐binding sites suggests origins of biological electron transfer. Sci Adv. 2022;8(2):eabj3984. 10.1126/sciadv.abj3984 35030025 PMC8759750

[pro70350-bib-0014] Cerdà‐Costa N , Xavier Gomis‐Rüth F . Architecture and function of metallopeptidase catalytic domains. Protein Sci. 2014;23(2):123–144. 10.1002/pro.2400 24596965 PMC3926739

[pro70350-bib-0015] Chai T , Draxler RR . Root mean square error (RMSE) or mean absolute error (MAE)?—arguments against avoiding RMSE in the literature. Geosci Model Dev. 2014;7(3):1247–1250. 10.5194/gmd-7-1247-2014

[pro70350-bib-0016] Chakrabarti P . Geometry of interaction of metal ions with histidine residues in protein structures. Protein Eng des Sel. 1990;4(1):57–63. 10.1093/protein/4.1.57 2290835

[pro70350-bib-0017] Cock PJA , Antao T , Chang JT , Chapman BA , Cox CJ , Dalke A , et al. Biopython: freely available python tools for computational molecular biology and bioinformatics. Bioinformatics. 2009;25(11):1422–1423. 10.1093/bioinformatics/btp163 19304878 PMC2682512

[pro70350-bib-0018] Daniel AG , Farrell NP . The dynamics of zinc sites in proteins: electronic basis for coordination sphere expansion at structural sites. Metallomics. 2014;6(12):2230–2241. 10.1039/C4MT00213J 25329367

[pro70350-bib-0020] Dudev T , Lim C . Competition among metal ions for protein binding sites: determinants of metal ion selectivity in proteins. Chem Rev. 2014;114(1):538–556. 10.1021/cr4004665 24040963

[pro70350-bib-0021] Dürr SL , Levy A , Rothlisberger U . Metal3D: a general deep learning framework for accurate metal ion location prediction in proteins. Nat Commun. 2023;14(1):2713. 10.1038/s41467-023-37870-6 37169763 PMC10175565

[pro70350-bib-0022] Feehan R , Copeland M , Franklin MW , Slusky JSG . MAHOMES II: a webserver for predicting if a metal binding site is enzymatic. Protein Sci. 2023;32(4):e4626. 10.1002/pro.4626 36916762 PMC10044107

[pro70350-bib-0067] Gane A , Bileschi ML , Dohan D , Speretta E , Héliou A , Meng‐Papaxanthos L , et al. ProtNLM: model‐based natural language protein annotation. 2022.

[pro70350-bib-0025] Hekkelman ML , de Vries I , Joosten RP , Perrakis A . AlphaFill: enriching AlphaFold models with ligands and cofactors. Nat Methods. 2023;20(2):205–213. 10.1038/s41592-022-01685-y 36424442 PMC9911346

[pro70350-bib-0026] Homma F , Huang J , van der Hoorn RAL . AlphaFold‐Multimer predicts cross‐kingdom interactions at the plant‐pathogen interface. Nat Commun. 2023;14(1):6040. 10.1038/s41467-023-41721-9 37758696 PMC10533508

[pro70350-bib-0027] Hopfner K‐P , Craig L , Moncalian G , Zinkel RA , Usui T , Owen BAL , et al. The Rad50 zinc‐hook is a structure joining Mre11 complexes in DNA recombination and repair. Nature. 2002;418(6897):562–566. 10.1038/nature00922 12152085

[pro70350-bib-0028] Jakeš J . A numerical method of fitting a multiparameter nonlinear function to experimental data in the $L_1$ norm. Appl Math. 1988;33(3):161–170. 10.21136/AM.1988.104299

[pro70350-bib-0031] Jumper J , Evans R , Pritzel A , Green T , Figurnov M , Ronneberger O , et al. Highly accurate protein structure prediction with AlphaFold. Nature. 2021;596(7873):583–589. 10.1038/s41586-021-03819-2 34265844 PMC8371605

[pro70350-bib-0032] Kim RS , Karin EL , Steinegger M . BFVD—a large repository of predicted viral protein structures. 2024 10.1101/2024.09.08.611582 PMC11701548 39574394

[pro70350-bib-0033] Laitaoja M , Valjakka J , Jänis J . Zinc coordination spheres in protein structures. Inorg Chem. 2013;52(19):10983–10991. 10.1021/ic401072d 24059258

[pro70350-bib-0034] Laveglia V , Bazayeva M , Andreini C , Rosato A . Hunting down zinc(II)‐binding sites in proteins with distance matrices. Bioinformatics. 2023;39(11):btad653. 10.1093/bioinformatics/btad653 37878807 PMC10630175

[pro70350-bib-0035] Levenberg K . A method for the solution of certain non‐linear problems in least squares. Quart Appl Math. 1944;2(2):164–168. 10.1090/qam/10666

[pro70350-bib-0036] Lever J , Krzywinski M , Altman N . Classification evaluation. Nat Methods. 2016;13(8):603–604. 10.1038/nmeth.3945

[pro70350-bib-0037] Li S , Hong M . Protonation, Tautomerization, and Rotameric structure of histidine: a comprehensive study by magic‐angle‐spinning solid‐state NMR. J Am Chem Soc. 2011;133(5):1534–1544. 10.1021/ja108943n 21207964 PMC4082993

[pro70350-bib-0038] Lin H‐T , Lin C‐J , Weng RC . A note on Platt's probabilistic outputs for support vector machines. Mach Learn. 2007;68(3):267–276. 10.1007/s10994-007-5018-6

[pro70350-bib-0039] Lin Z , Akin H , Rao R , Hie B , Zhu Z , Lu W , et al. Evolutionary‐scale prediction of atomic‐level protein structure with a language model. Science. 2023;379(6637):1123–1130. 10.1126/science.ade2574 36927031

[pro70350-bib-0040] Maret W . Metalloproteomics, metalloproteomes, and the annotation of metalloproteins. Metallomics. 2010;2(2):117–125. 10.1039/B915804A 21069142

[pro70350-bib-0041] Maret W . Zinc in cellular regulation: the nature and significance of ‘zinc signals’. Int J Mol Sci. 2017;18(11):2285. 10.3390/ijms18112285 29088067 PMC5713255

[pro70350-bib-0042] McCall KA , Huang C , Fierke CA . Function and mechanism of zinc Metalloenzymes. J Nutr. 2000;130(5):1437S–1446S. 10.1093/jn/130.5.1437S 10801957

[pro70350-bib-0043] Niculescu‐Mizil A , Caruana R . Predicting good probabilities with supervised learning. Proceedings of the 22nd international conference on machine learning—ICML ‘05. Bonn, Germany: ACM Press; 2005. p. 625–632. 10.1145/1102351.1102430

[pro70350-bib-0044] Nomburg J , Doherty EE , Price N , Bellieny‐Rabelo D , Zhu YK , Doudna JA . Birth of protein folds and functions in the virome. Nature. 2024;633(8030):710–717. 10.1038/s41586-024-07809-y 39187718 PMC11410667

[pro70350-bib-0045] Platt J . Probabilistic outputs for support vector machines and comparisons to regularized likelihood methods. Advances in large margin classifiers. 1999;10( 3):61–74.

[pro70350-bib-0046] Ruszkowski M , Dauter Z . Structures of Medicago truncatula L‐Histidinol dehydrogenase show rearrangements required for NAD+ binding and the cofactor positioned to accept a hydride. Sci Rep. 2017;7(1):10476. 10.1038/s41598-017-10859-0 28874718 PMC5585171

[pro70350-bib-0047] Saldaño T , Escobedo N , Marchetti J , Zea DJ , Mac Donagh J , Velez Rueda AJ , et al. Impact of protein conformational diversity on AlphaFold predictions. Bioinformatics. 2022;38(10):2742–2748. 10.1093/bioinformatics/btac202 35561203

[pro70350-bib-0048] Sendra KM , Barwinska‐Sendra A , Mackenzie ES , Baslé A , Kehl‐Fie TE , Waldron KJ . An ancient metalloenzyme evolves through metal preference modulation. Nat Ecol Evol. 2023;7(5):732–744. 10.1038/s41559-023-02012-0 37037909 PMC10172142

[pro70350-bib-0049] Shapovalov MV , Dunbrack RL . A smoothed backbone‐dependent Rotamer library for proteins derived from adaptive kernel density estimates and regressions. Structure. 2011;19(6):844–858. 10.1016/j.str.2011.03.019 21645855 PMC3118414

[pro70350-bib-0050] Shenoy A , Kalakoti Y , Sundar D , Elofsson A . M‐ionic: prediction of metal‐ion‐binding sites from sequence using residue embeddings. Bioinformatics. 2024;40(1):btad782. 10.1093/bioinformatics/btad782 38175787 PMC10792727

[pro70350-bib-0051] Shishir FS , Sarker B , Rahman F , Shomaji S . MetaLLM: residue‐wise metal ion prediction using deep transformer model. bioRxiv. 2023. 10.1101/2023.03.20.533488

[pro70350-bib-0052] Tamames B , Sousa SF , Tamames J , Fernandes PA , Ramos MJ . Analysis of zinc‐ligand bond lengths in metalloproteins: trends and patterns. Proteins. 2007;69(3):466–475. 10.1002/prot.21536 17623850

[pro70350-bib-0053] Theobald DL . Rapid calculation of RMSDs using a quaternion‐based characteristic polynomial. Acta Crystallogr A Found Crystallogr. 2005;61(4):478–480. 10.1107/S0108767305015266 15973002

[pro70350-bib-0054] Varadi M , Anyango S , Deshpande M , Nair S , Natassia C , Yordanova G , et al. AlphaFold protein structure database: massively expanding the structural coverage of protein‐sequence space with high‐accuracy models. Nucleic Acids Res. 2022;50(D1):D439–D444. 10.1093/nar/gkab1061 34791371 PMC8728224

[pro70350-bib-0055] Varadi M , Bertoni D , Magana P , Paramval U , Pidruchna I , Radhakrishnan M , et al. AlphaFold protein structure database in 2024: providing structure coverage for over 214 million protein sequences. Nucleic Acids Res. 2024;52(D1):D368–D375. 10.1093/nar/gkad1011 37933859 PMC10767828

[pro70350-bib-0056] Waldron KJ , Robinson NJ . How do bacterial cells ensure that metalloproteins get the correct metal? Nat Rev Microbiol. 2009;7(1):25–35. 10.1038/nrmicro2057 19079350

[pro70350-bib-0057] Weiss A , Murdoch CC , Edmonds KA , Jordan MR , Monteith AJ , Perera YR , et al. Zn‐regulated GTPase metalloprotein activator 1 modulates vertebrate zinc homeostasisc. Cell. 2022;185(12):2148–2163.e27. 10.1016/j.cell.2022.04.011 35584702 PMC9189065

[pro70350-bib-0059] Yang T‐Y , Dudev T , Lim C . Mononuclear versus binuclear metal‐binding sites: metal‐binding affinity and selectivity from PDB survey and DFT/CDM calculations. J Am Chem Soc. 2008;130(12):3844–3852. 10.1021/ja076277h 18303888

[pro70350-bib-0060] Yao S , Flight RM , Rouchka EC , Moseley HNB . A less‐biased analysis of metalloproteins reveals novel zinc coordination geometries. Proteins. 2015;83(8):1470–1487. 10.1002/prot.24834 26009987 PMC4539273

[pro70350-bib-0061] Ye N , Zhou F , Liang X , Chai H , Fan J , Li B , et al. A comprehensive review of computation‐based metal‐binding prediction approaches at the residue level. Biomed Res Int. 2022;2022:1–19. 10.1155/2022/8965712 PMC8989566 35402609

[pro70350-bib-0068] Yeo J, Han Y , Bordin N , Lau AM , Kandathil SM , Kim H , et al. Metagenomic‐scale analysis of the predicted protein structure universe. bioRxiv. 2025.

[pro70350-bib-0063] Yuan Q , Chen S , Wang Y , Zhao H , Yang Y . Alignment‐free metal ion‐binding site prediction from protein sequence through pretrained language model and multi‐task learning. Brief Bioinform. 2022;23(6):bbac444. 10.1093/bib/bbac444 36274238

[pro70350-bib-0064] Zhang H‐Q , Liu SH , Li R , Yu JW , Ye DX , Yuan SS , et al. MIBPred: ensemble learning‐based metal ion‐binding protein classifier. ACS Omega. 2024;9(7):8439–8447. 10.1021/acsomega.3c09587 38405489 PMC10882704

[pro70350-bib-0065] Zhang Y , Zheng J . Bioinformatics of Metalloproteins and Metalloproteomes. Molecules. 2020;25(15):3366. 10.3390/molecules25153366 32722260 PMC7435645

[pro70350-bib-0066] Zheng H , Zhong J , Gucwa M , Zhang Y , Ma H , Deng L et al. PinMyMetal: a hybrid learning system to accurately model metal binding sites in macromolecules. Nat Commun. 2025;16(1):3043. 10.21203/rs.3.rs-3908734/v1 40155596 PMC11953438

