# Supplementary material for: Interpretable prediction of zinc ion location in proteins with ZincSight
Source: Protein Sci. 2025 Oct 30;34(11):e70350. doi: 10.1002/pro.70350 (PMC12572635; doi:10.1002/pro.70350)
Supplement: Supplementary file 1 — Table S1: Breakdown of the 117 false‐positive predictions by ZincSight (p ≥ 0.5) on the coverage evaluation set of 830 x‐ray crystal structures (≤ 2.5 Å; 1588 true Zn2+ sites). Categories: copper‐binding, iron‐binding, calcium site annotated as zinc‐binding site, low‐occupancy zinc‐binding sites (occupancy < 0.5), predictions with only two actual ligation residues, UniProt‐annotated zinc‐binding sites lacking zinc ion in the structure, and other cases. Table S3: ZincSight predictions (p > 0.5) for 200 randomly selected representatives of the AFESM “only ESM” clusters. Table S4: A list of the amino acids and specific atoms known to mediate zinc ion coordination (Bowman et al., 2016; Dudev & Lim, 2014). ZincSight considers these residues and the specified atoms. Table S5: Test set 4; the AlphaFold2 test set of protein structures used for evaluating zinc‐binding site predictions. The table provides AlphaFold ID, PDB ID, UniProt ID, and Pfam ID for each protein. Figure S2: Distribution of the RMSD values of the predicted zinc‐binding sites of the AF2 test set (test set 4) relative to the corresponding true PDB‐solved sites. The analysis targeted specific regions of the PDB structures encompassing zinc‐binding residues and their adjacent three‐residue sequence neighbors, superimposing these onto corresponding regions of the AF2 models. The mean RMSD was 0.60 Å, indicating a high degree of structural similarity. The variance of 0.64 Å is skewed by two sites with considerably high RMSD values (~4 Å). These higher RMSD values correspond to AF2 structures where parts of their zinc‐binding residues are misoriented relative to the superimposed PDB structure. Despite this, the vast majority of RMSD values are below 1 Å. This structural similarity highlights the accuracy of AF2 models, particularly within zinc‐binding site regions, enabling ZincSight to reliably predict most zinc‐binding sites in this set. Figure S3: PR in identifying zinc‐binding sites within 5 Å in test set 1. [file PRO-34-e70350-s001.docx]

## Supplementary information

| **Classification** | **Number of sites** | **Percentage** | **PDB IDs** |
| --- | --- | --- | --- |
| Copper binding | 61 | 52.14% | 7TIH, 7W3E, 7WWY, 7XMA, 7YPR, 8GSQ, 8H8R, 8H8S, 8IJN, 8IMD, 8IQ1, 8JD8, 8JD8, 9IKG, 9KUM, 6ZS1 |
| Iron binding | 16 | 13.68% | 8AXY, 8BRN, 8DZK, 8RLY |
| Calcium binding | 1 | 0.85% | 7G2T |
| Occupancy < 0.5 | 11 | 9.40% | 7O45, 7OEZ, 7PVA,7SBJ, 8VKT, 9C3D, 8F1B |
| Two residues bind Zinc | 10 | 8.55% | 7TM9, 8B9N, 8RHI, 8RZU, 8SNK, 8VC5, 7F0U |
| annotated in UniProt as zinc-binding | 8 | 6.84% | 7XA4, 7ZVY, 9FYL |
| Other | 10 | 8.55% | 7D1D, 7CM0, 7CJG, 7P99, 7T09, 7YGI, 8CO4 |

**Table S1:** Breakdown of the 117 false-positive predictions by ZincSight (p ≥ 0.5) on the coverage evaluation set of 830 X-ray crystal structures (≤ 2.5 Å; 1,588 true Zn²⁺ sites). Categories: copper-binding, iron-binding, calcium site annotated as zinc-binding site, low-occupancy zinc-binding sites (occupancy < 0.5), predictions with only two actual ligation residues, UniProt-annotated zinc-binding sites lacking zinc ion in the structure, and other cases.

**Table S2 (attached Excel sheet)**: Residue-level details of the 117 false-positive predictions summarized in Table S1, listing the predicted zinc-binding residues for each case.

| **structure id** | **chain resi 1** | **chain resi 2** | **chain resi 3** | **chain resi 4** | **Resi comb ordered** | **Predicted ion pos** | **prob** |
| --- | --- | --- | --- | --- | --- | --- | --- |
| MGYP000663010549 | A_47 | A_24 | A_26 | A_49 | {C,C,C,H} | [1.51, 0.59, -1.40] | 0.982 |
| MGYP001596489143 | A_73 | A_85 | A_71 | A_87 | {C,C,C,H} | [9.30, 5.26, -5.05] | 0.965 |
| MGYP002623597247 | A_10 | A_32 | A_7 | A_35 | {C,C,C,C} | [5.36, 3.66, -8.77] | 0.962 |
| MGYP001130174518 | A_49 | A_85 | A_88 | A_54 | {C,C,C,C} | [3.30, 14.13, -12.49] | 0.952 |
| MGYP002639704763 | A_10 | A_28 | A_31 | A_13 | {C,C,C,C} | [23.78, 42.93, -45.66] | 0.92 |
| MGYP001130174518 | A_121 | A_99 | A_104 | A_124 | {C,C,C,C} | [14.33, -5.02, -12.36] | 0.849 |
| MGYP001351529692 | A_168 | A_172 | A_178 |  | {H,H,H} | [8.09, -7.23, -0.98] | 0.845 |
| MGYP000607038021 | A_112 | A_210 | A_110 |  | {D,H,H} | [-8.59, 1.85, -2.86] | 0.84 |
| MGYP000977293256 | A_101 | A_126 | A_129 |  | {C,C,H} | [ -7.08, -6.06, 12.97] | 0.823 |
| MGYP001101007251 | A_368 | A_371 | A_345 |  | {C,C,H} | [-14.99, -6.41, 14.48] | 0.821 |
| MGYP005969600693 | A_441 | A_427 | A_424 | A_438 | {C,C,C,C} | [-13.75, 15.85, -43.97] | 0.811 |
| MGYP003538412071 | A_6 | A_33 | A_32 |  | {D,H,H} | [1.87, -4.76, 5.74] | 0.795 |
| MGYP003324896311 | A_114 | A_111 | A_196 |  | {E,H,H} | [6.57, -1.10, -6.41] | 0.735 |
| MGYP001222855543 | A_105 | A_92 | A_88 |  | {E,H,H} | [7.75, -3.78, 6.11] | 0.726 |
| MGYP000386160404 | A_27 | A_13 | A_16 | A_30 | {C,C,C,C} | [7.60, 17.94, -31.49] | 0.723 |
| MGYP001797524625 | A_78 | A_19 | A_35 |  | {E,H,H} | [-2.77, -8.09, -3.79] | 0.708 |
| MGYP003538412071 | A_31 | A_33 | A_32 |  | {D,H,H} | [3.75, -7.32, 8.43] | 0.665 |
| MGYP001488395935 | A_238 | A_242 | A_291 |  | {D,H,D} | [10.02, 20.74, -12.31] | 0.547 |
| MGYP001488395935 | A_291 | A_242 | A_276 |  | {D,H,E} | [10.66, 20.54, -9.47] | 0.527 |

**Table S3:** ZincSight predictions (p > 0.5) for 200 randomly selected representatives of the AFESM “only ESM” clusters.

| **Residue Name** | **Zinc-Binding Atoms** |
| --- | --- |
| **His** | ND1, NE2 |
| **Glu** | OE1, OE2 |
| **Cys** | SG |
| **Asp** | OD1, OD2 |
| **Thr** | OG1 |
| **Tyr** | OH |
| **Ser** | OG |
| **Asn** | OD1 |

**Table S4:** A list of the amino acids and specific atoms known to mediate zinc ion coordination [1], [2]. ZincSight considers these residues and the specified atoms.

| **AlphaFold ID** | **UniProt ID** | **Pfam ID** | **PDB ID** |
| --- | --- | --- | --- |
| AF-Q08281-F1-v4 | Q08281 | PF17120 | 8ADL |
| AF-P38164-F1-v4 | P38164 | PF17034 | 8ADL |
| AF-Q86T03-F1-v4 | Q86T03 | PF09788 | 8OQH |
| AF-Q5W0Q7-F1-v4 | Q5W0Q7 | PF15499 | 7P99 |
| AF-Q9NUN7-F1-v4 | Q9NUN7 | PF05875 | 6YXH |
| AF-G8ZFK7-F1-v4 | G8ZFK7 | PF07754 | 7ZHG |
| AF-Q66K64-F1-v4 | Q66K64 | PF14939 | 6PAI |
| AF-Q03760-F1-v4 | Q03760 | PF07967 | 7RDN |
| AF-Q7MVV4-F1-v4 | Q7MVV4 | PF08665 | 7PVA |
| AF-A0A3F2YM30-F1-v4 | A0A3F2YM30 | PF13391 | 6GHS |
| AF-A0A068N621-F1-v4 | A0A068N621 | PF18921 | 7TXU |
| AF-P0A6G5-F1-v4 | P0A6G5 | PF03802 | 7DCM |
| AF-A0A5H1ZR49-F1-v4 | A0A5H1ZR49 | PF13582 | 6O38 |
| AF-Q9NXF7-F1-v4 | Q9NXF7 | PF15349 | 8G46 |
| AF-Q9BWG6-F1-v4 | Q9BWG6 | PF15803 | 7DVQ |
| AF-Q2UFA9-F1-v4 | Q2UFA9 | PF18785 | 7DRY |
| AF-Q6CXX6-F1-v4 | Q6CXX6 | PF04376 | 7WG4 |
| AF-Q9D1N4-F1-v4 | Q9D1N4 | PF12036 | 8T03 |
| AF-A0A292DHH8-F1-v4 | A0A292DHH8 | PF10460 | 7BB8 |
| AF-A0A0F6AZI6-F1-v4 | A0A0F6AZI6 | PF07108 | 6GGO |
| AF-Q8N8R7-F1-v4 | Q8N8R7 | PF14949 | 8HFP |
| AF-Q9M1V3-F1-v4 | Q9M1V3 | PF14432 | 7O4E |
| AF-Q9KP27-F1-v4 | Q9KP27 | PF10986 | 8F1B |
| AF-Q68EN5-F1-v4 | Q68EN5 | PF08014 | 7Z5H |
| AF-Q2K0Z2-F1-v4 | Q2K0Z2 | PF06089 | 7OZ6 |
| AF-A0A2U3D0N8-F1-v4 | A0A2U3D0N8 | PF07282 | 8DZJ |
| AF-Q8NBJ9-F1-v4 | Q8NBJ9 | PF13965 | 7Y63 |

**Table S5:** Test set 4; the AlphaFold2 test set of protein structures used for evaluating zinc-binding site predictions. The table provides AlphaFold ID, PDB ID, UniProt ID, and Pfam ID for each protein.

**Figure S1: Validation of predicted probabilities for zinc ion binding site prediction.** (a) The distribution of scores assigned to true zinc ion binding sites (blue) and false candidate sites (orange). True scores follow a Gaussian-like distribution with distinct separation from false scores. (b) The distribution of Platt scaling [5] calibrated probabilities for true zinc ion binding sites (blue) and false candidates (orange). Calibration reduces variance and highlights clearer separation between groups. (c) Calibration curve showing close alignment between predicted probabilities and observed frequencies for zinc ion binding. Calibration performance is summarized by a Brier score of 0.0346 [6] and a log loss of 0.1163, where lower values indicate better calibration.


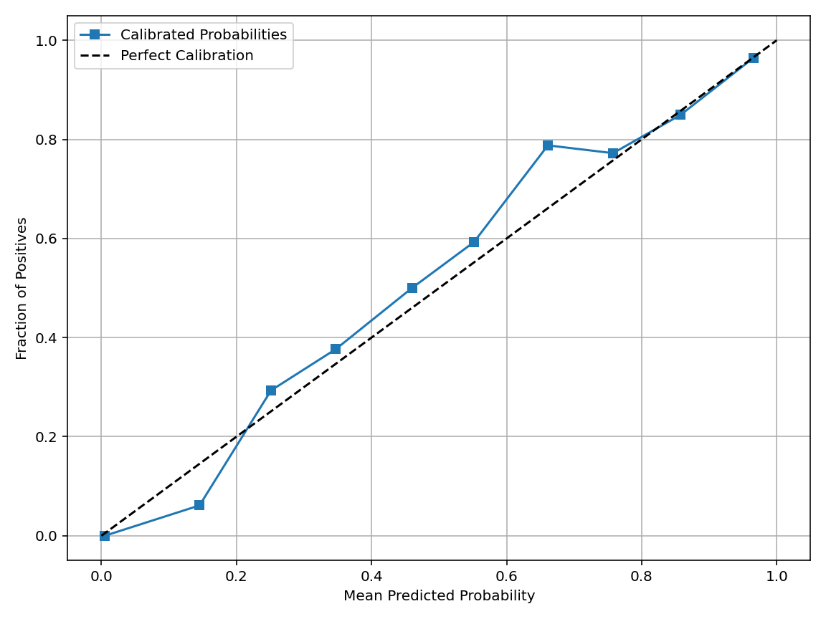

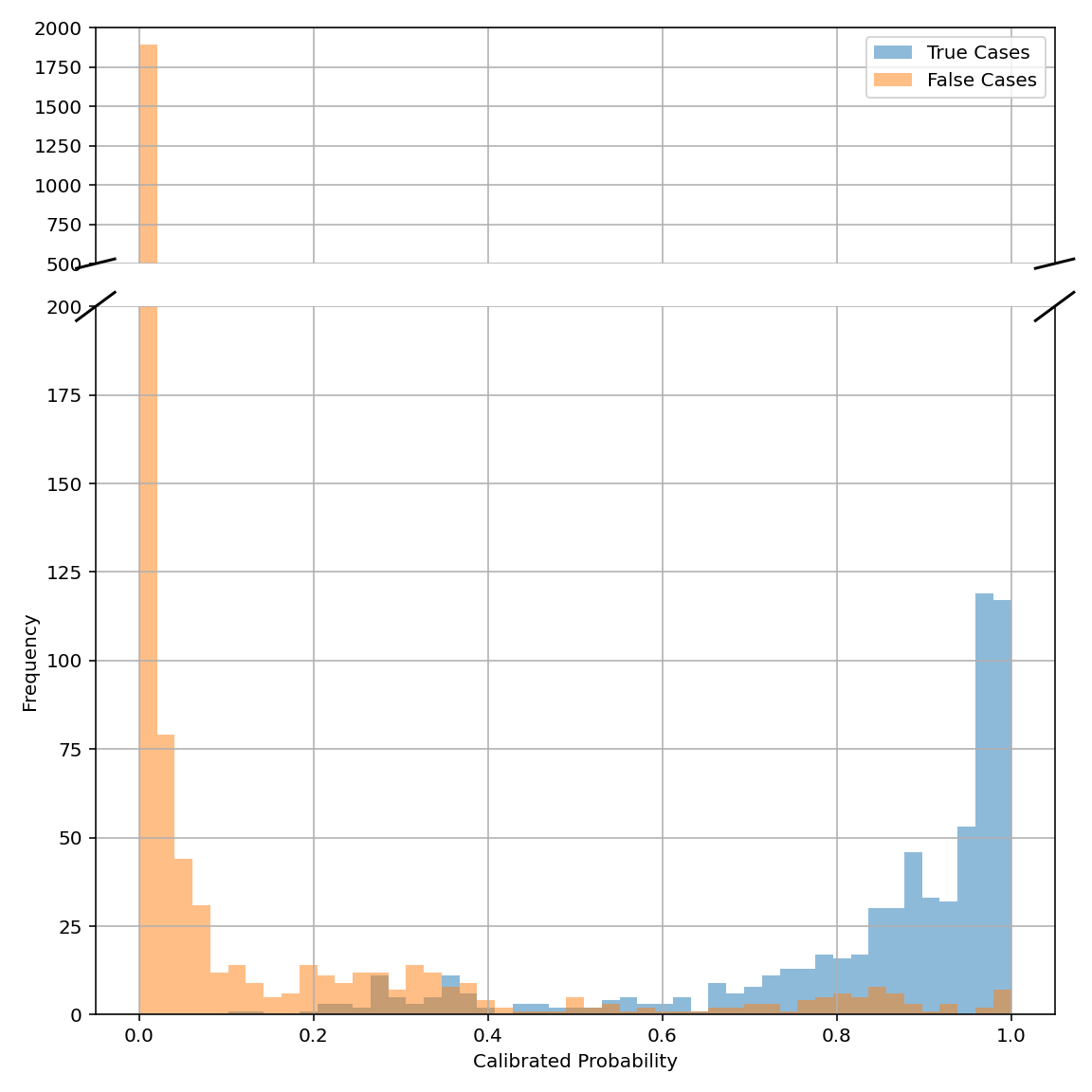

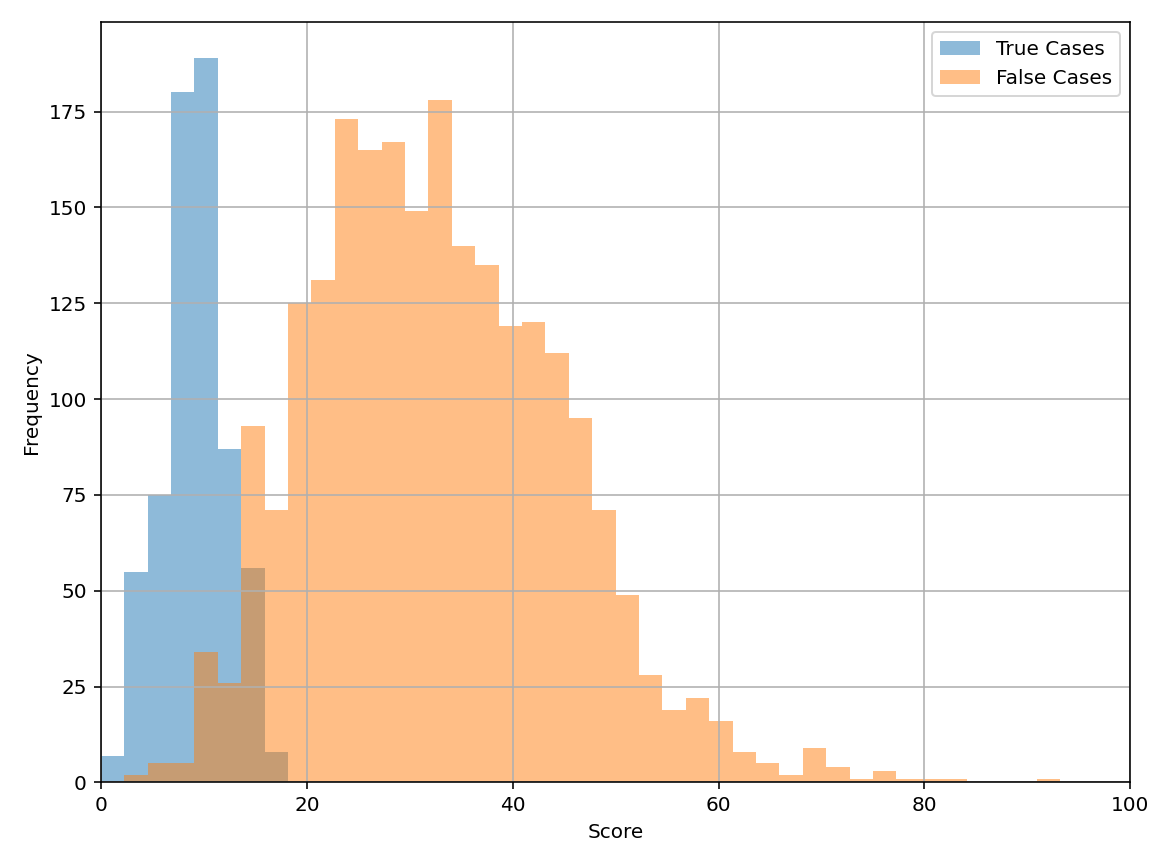


**a)**

**b)**

**c)**


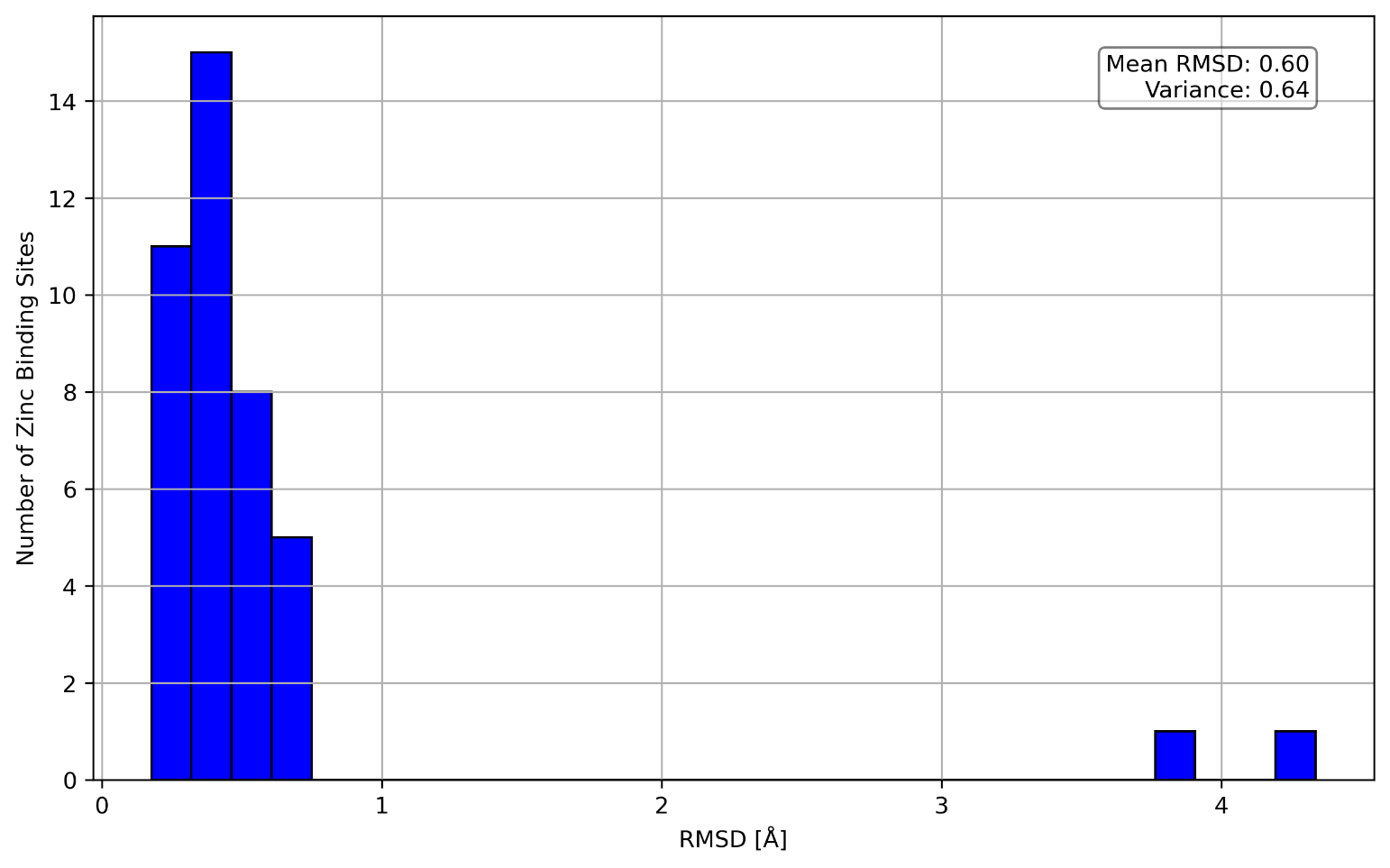


**Figure S2: Distribution of the RMSD values of the predicted zinc-binding sites of the AF2 test set (test set 4) relative to the corresponding true PDB-solved sites.** The analysis targeted specific regions of the PDB structures encompassing zinc-binding residues and their adjacent three-residue sequence neighbors, superimposing these onto corresponding regions of the AF2 models. The mean RMSD was 0.60 Å, indicating a high degree of structural similarity. The variance of 0.64 Å is skewed by two sites with considerably high RMSD values (~4 Å). These higher RMSD values correspond to AF2 structures where parts of their zinc-binding residues are misoriented relative to the superimposed PDB structure. Despite this, the vast majority of RMSD values are below 1 Å. This structural similarity highlights the accuracy of AF2 models, particularly within zinc-binding site regions, enabling ZincSight to reliably predict most zinc-binding sites in this set.


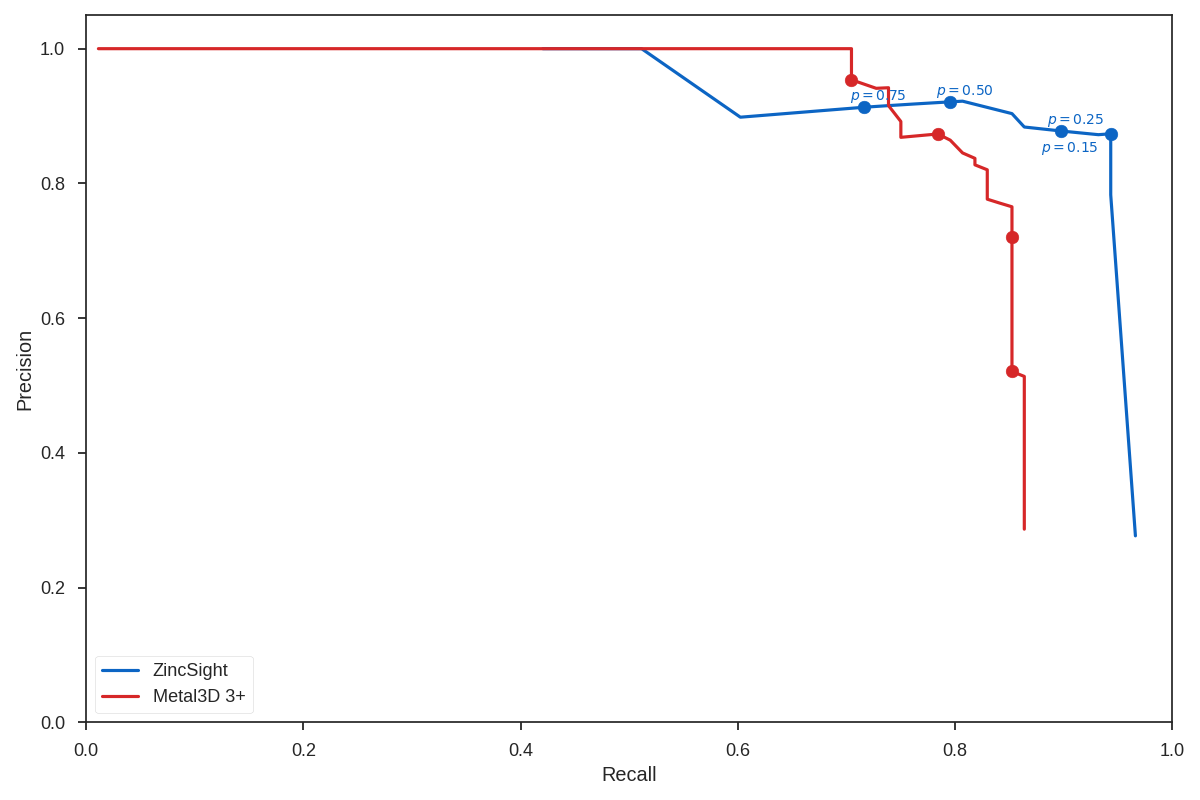


**Figure S3: PR in identifying zinc-binding sites within 5 Å in test set 1.** PR for ZincSight (blue curve) and Metal3D (red curve) predictions for all 86 “3+ zinc sites” of the test set of 59 proteins structures. The probability thresholds $p$ used for predictions are marked. ZincSight maintains high precision across different recall values. Metal3D has a somewhat higher precision in recall region ~0.5-through-~0.75, and ZincSight has higher precision above recall=~0.75.


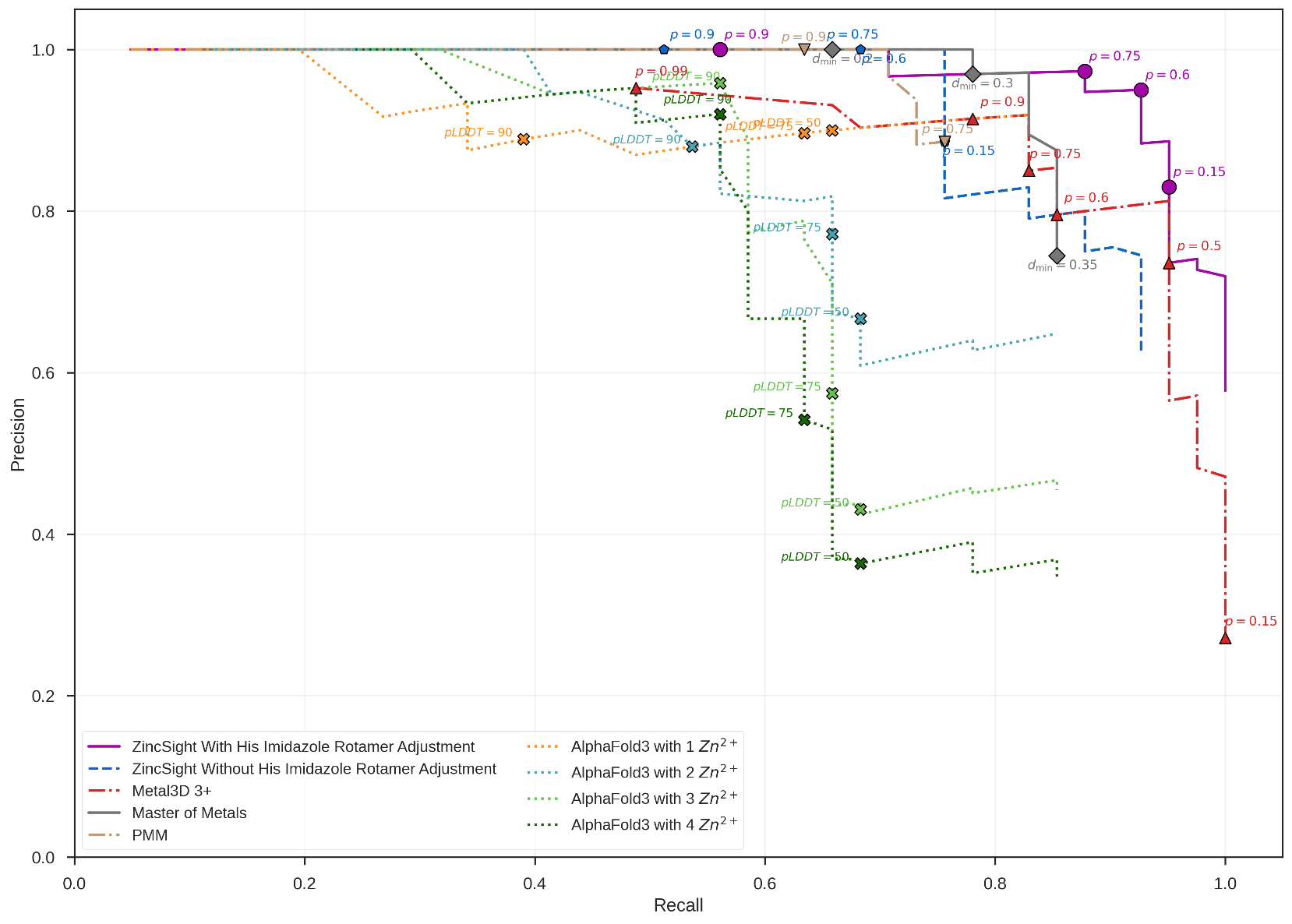


**Figure S4: PR in identifying zinc-binding sites within 5 Å in test set 4 (AF2 models).** The PR curves for ZincSight, Metal3D, MoM, PMM, and AF3 prediction tasks for zinc-binding sites within this test set. PR metrics for ZincSight predictions were computed for the prediction task with (purple curve) and without (blue curve) optimization of the rotameric state of the histidine imidazole. The ground truth zinc ions coordinates are determined by superimposing zinc-binding sites from each model's corresponding PDB structure, sharing the same UniProt accession, onto the AF2 model. Looking at the full spectrum, ZincSight is on par with, perhaps slightly better then, the best alternatives.


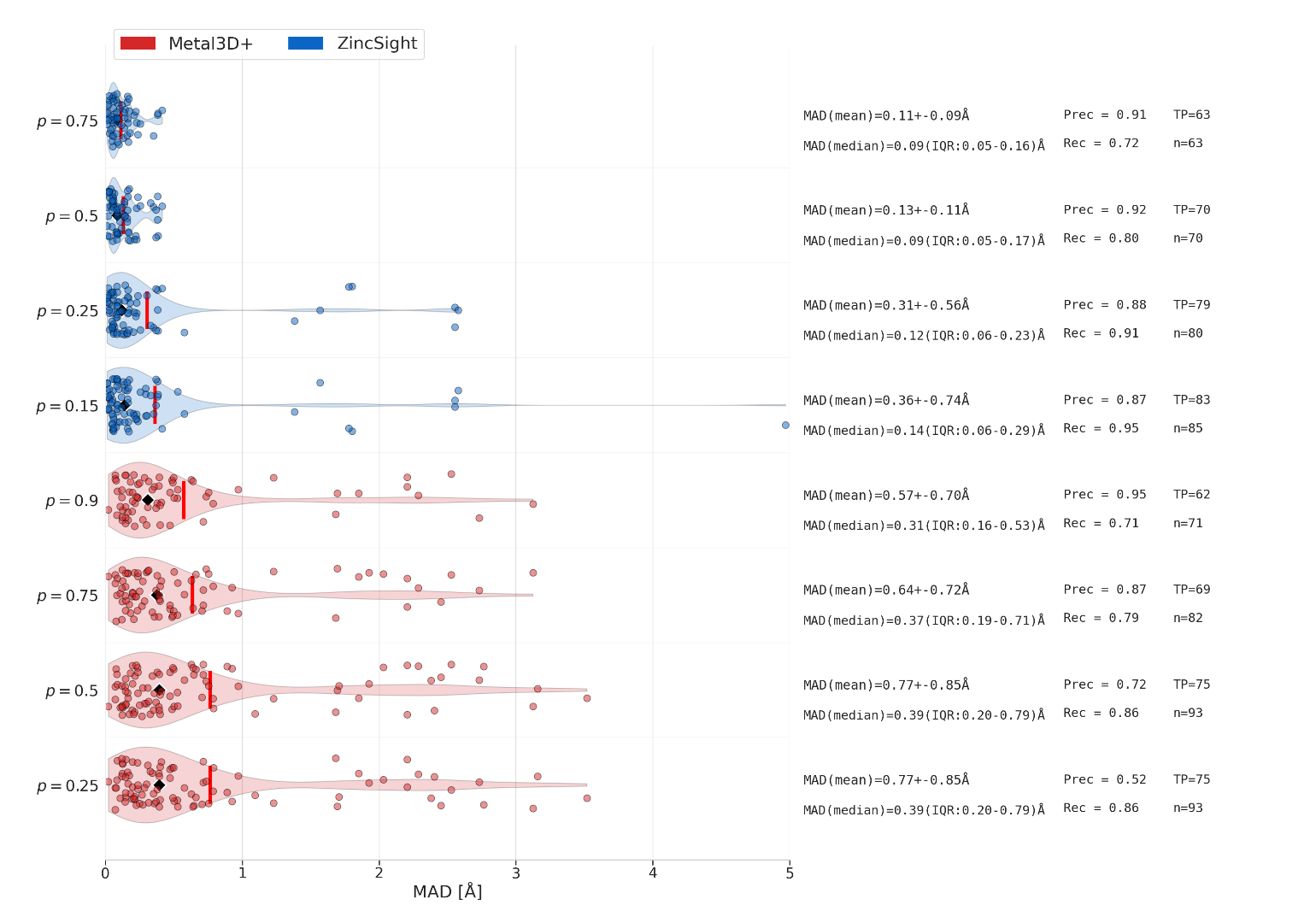


**Figure S5: MAD for correctly predicted sites within 5 Å in test set 1.** Calculated MAD values for all 86 “3+ zinc sites” in the Metal3D test set that were correctly predicted by ZincSight (blue) and Metal3D (red). ZincSight’s MAD values measured at set of predicted probabilities thresholds ranging from 0.75 to 0.15. Per each probability threshold, *n* is the number of the overall predicted metal sites within 5 Å of the actual zinc position. TP is the number of correctly predicted sites (multiple predicted sites can be within 5 Å radius from a single TP site). Each dot represents the measured distance (in Å) between a predicted metal ion position and the actual zinc ion position. The violin shape shows the kernel density of these distances, with the red vertical line marking the mean and a black diamond shape marking the median. We see that for different thresholds, the MAD values of ZincSight are smaller than those of Metal3D predictions.

**
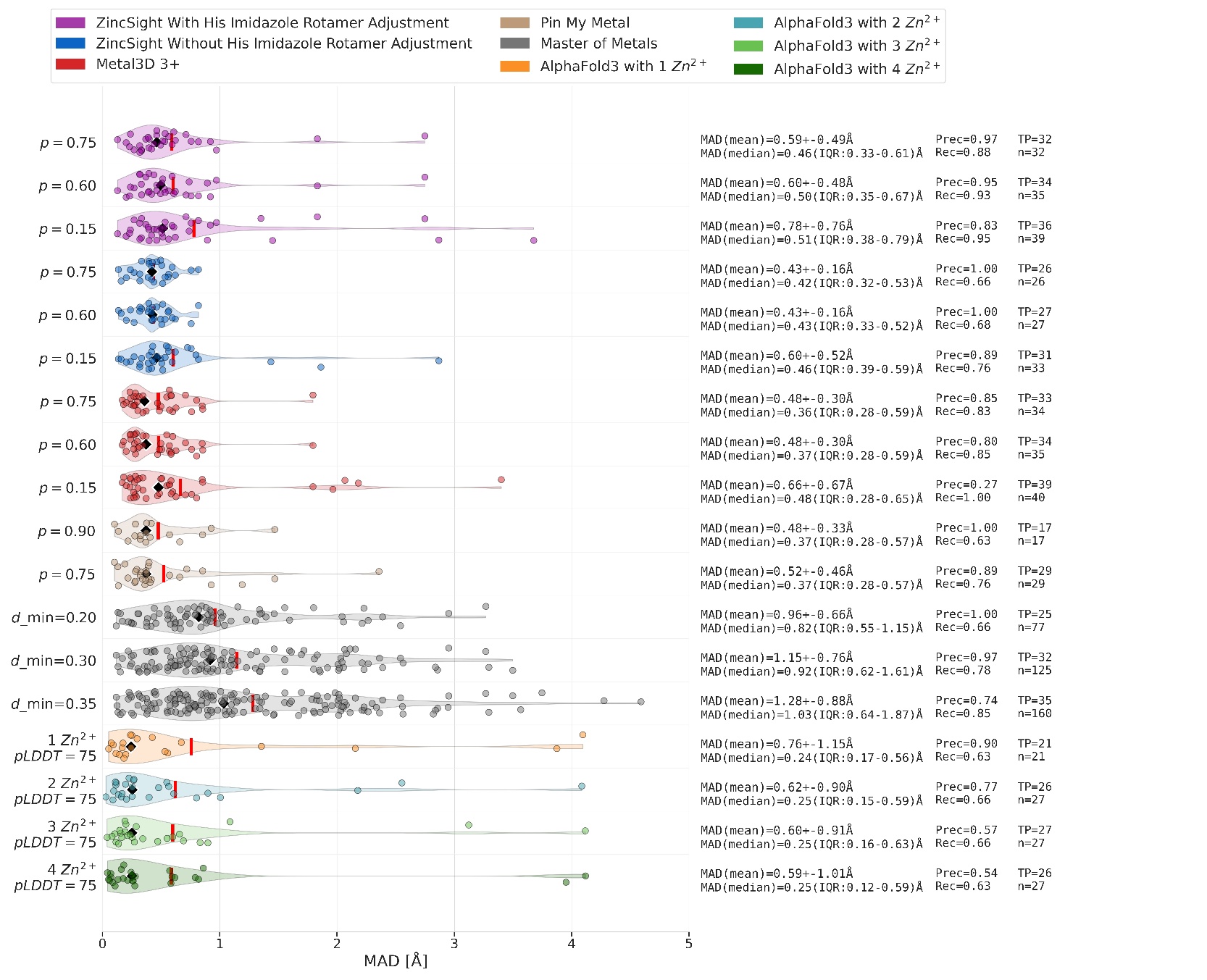
Figure S6: MAD for correctly predicted sites within 5 Å on test set 4 (the AF models).** Calculated MAD for all zinc-binding sites within the AF test set that ZincSight, Metal3d, MoM, PMM and AF3 correctly predicted. MAD values were measured at probabilities estimates threshold of$0.75, 0.60 \mathrm{and}0.15$for ZincSight analyses with and without rotations. For Metal3D 3+ (red) we used the same probability estimates thresholds. For AF3 we considered a pLDDT threshold of 75 for each of the predicted zinc ions obtained by query with stoichiometry of one-through-four zinc ions per protein. For each predicted probability threshold, *n* represents the number of the overall predicted metal sites within 5 Å of the actual zinc position. The number of the actual predicted sites is represented by TP (multiple predicted sites can be within 5 Å radius from a single TP sites).  Each dot represents the measured distance (in Å) between a predicted metal ion position and the actual zinc ion position. The violin shape shows the kernel density of these distances, with the red vertical line marking the mean and a black diamond shape marking the median.


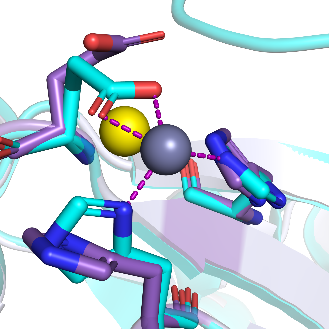

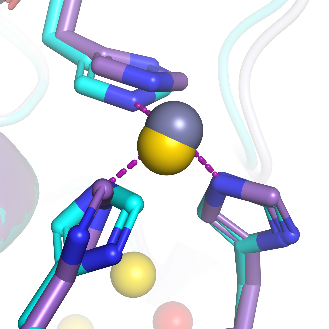

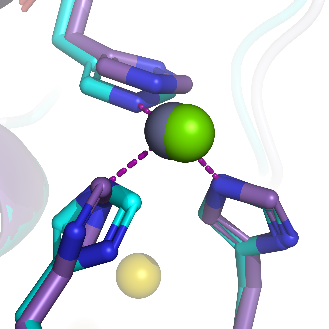

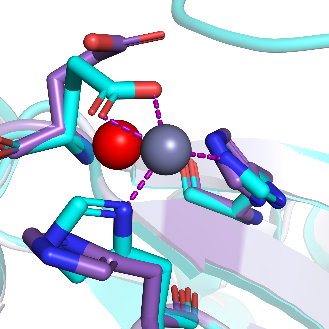


(d)

(c)

(b)

(a)


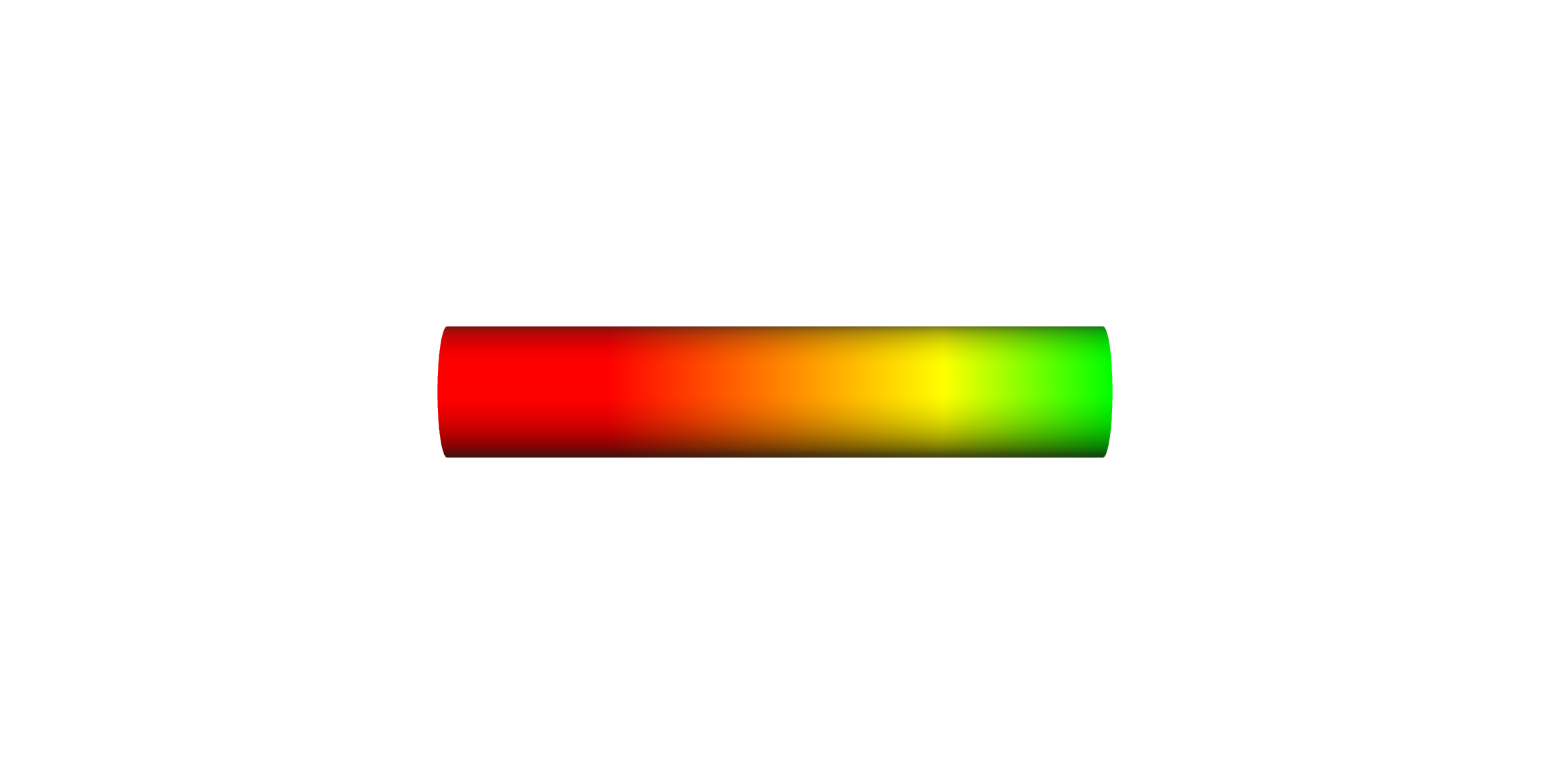

$$p\approx0\%$$

$$p\approx100\%$$

**Figure S7: Comparison of zinc ion predictions in example AF models with and without His rotamer sampling.** As an example of predictions in AF models, we show the AF2 prediction (purple) of *Homo sapiens* SIDT2 protein (AF ID: AF- Q8NBJ9-F1) (a, b) and *Vibrio cholerae* ZrgA protein (c, d) (AF ID: AF-Q9KP27-F1), superimposed with their corresponding experimental PDB structures (cyan) (IDs: 7Y63 and 8F1B, respectively). Predictions are shown with (a, c) and without (b, d) rotamer sampling. Experimentally determined zinc ions (dark-gray spheres) from PDB structures are shown for reference. Predicted zinc ion locations are colored according to their ZincSight-assigned predicted probabilities. Probability colorbar is provided, ranging from red (unfavorable, low probability), through yellow, to green (favorable, high probability). Implementation of rotamer sampling led to more favorable scores for zinc ion binding sites in both SIDT2 (green sphere, a) and ZrgA (yellow sphere, c) compared to predictions without rotamer sampling (orange and red spheres in b and d, respectively). In this example, ZincSight demonstrates its ability to predict zinc ion locations even when AF models' histidine imidazole group is initially misoriented.


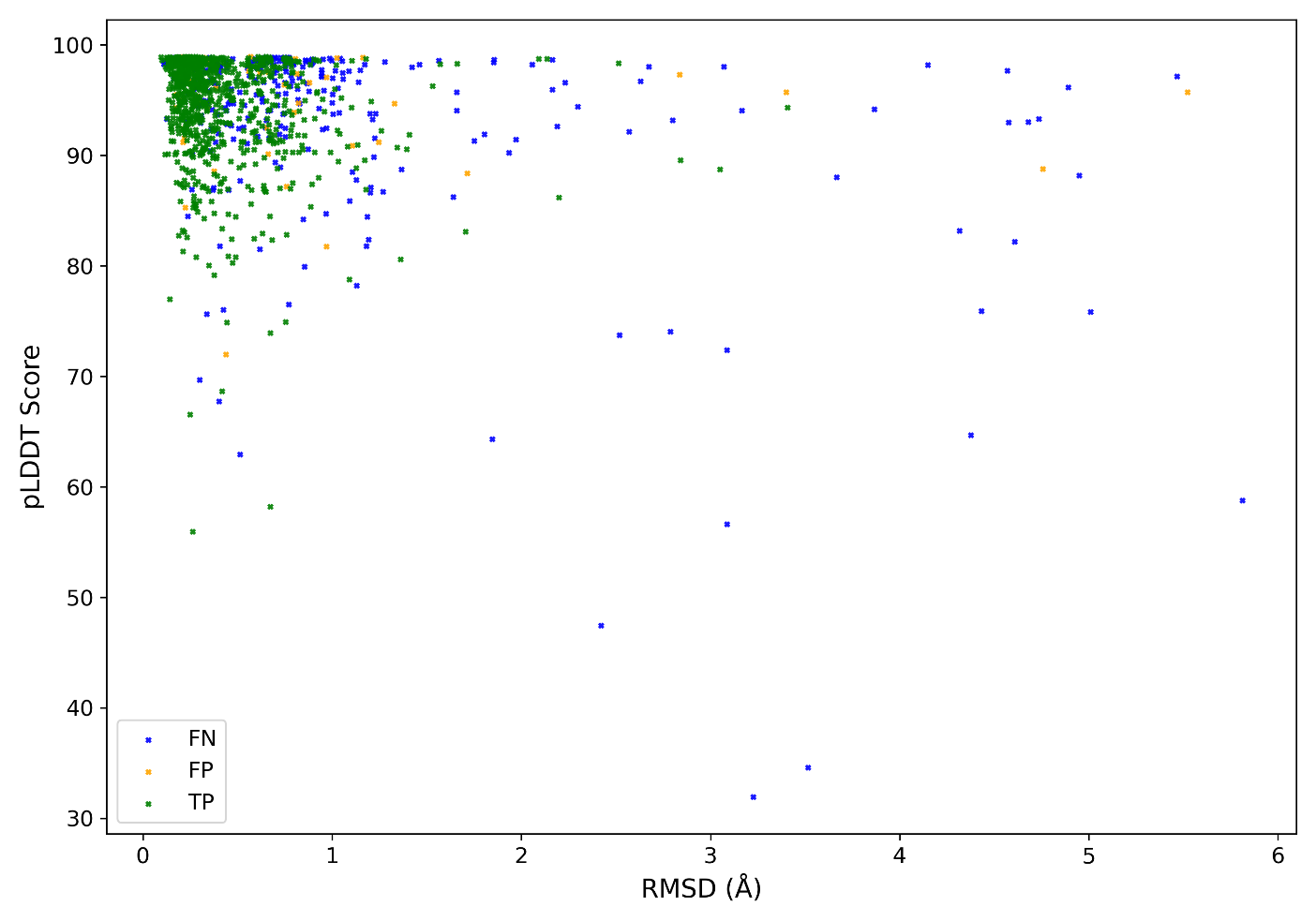


**Figure S8. Correlation between AF2 mean pLDDT and structural similarity of predicted zinc‑binding sites to their experimental counterparts.** Scatter plot of mean pLDDT versus RMSD between PDB‑validated zinc‑coordinating residues and their AF2‑predicted counterparts for each site (test set 5). Points are colored by outcome: TP (green) have the predicted zinc ion within 2 Å of the experimental ion upon superposition; FP (orange) lack any PDB zinc within 2 Å; FN (blue) are experimental sites with no prediction within 2 Å. Zinc-binding sites located in regions with mean pLDDT below 80 display lower structural accuracy. ZincSight is much more likely to overlook these compared to sites in regions assigned pLDDT above 80.


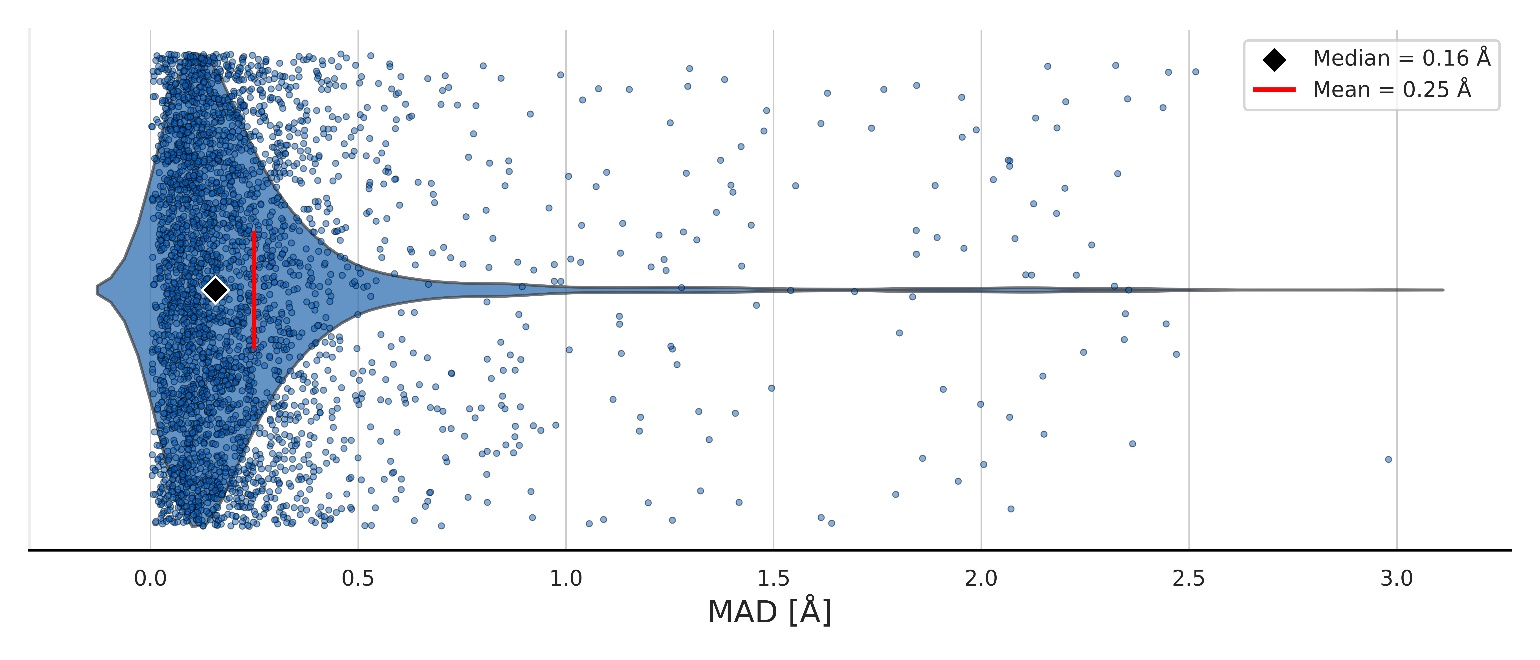


**Figure S9: Distance between predicted and actual zinc ion location within the training set.** MAD of distances between predicted zinc ion locations in correctly predicted binding sites, within the training set, to actual zinc ion location. Only sites for which all zinc-binding residues have been correctly predicted, have been taken to account in this analysis. The small MAD values attest to the accuracy of ZincSight in placing the zinc ions near their real positions. The violin shape shows the kernel density of the distances, with the red vertical line marking the mean and a black diamond shape marking the median.

**b)**

**c)**

**d)**


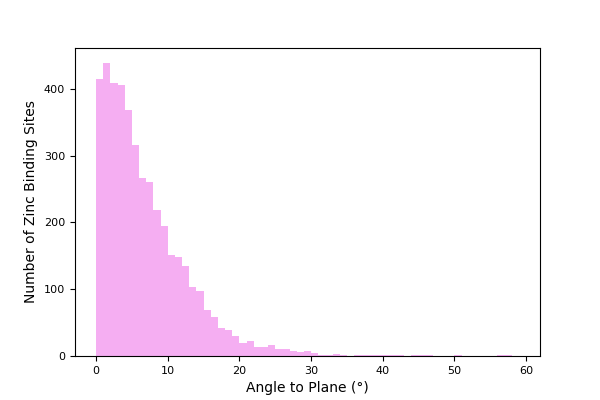

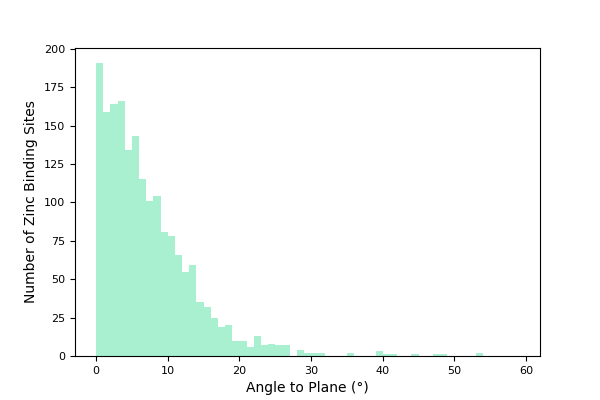

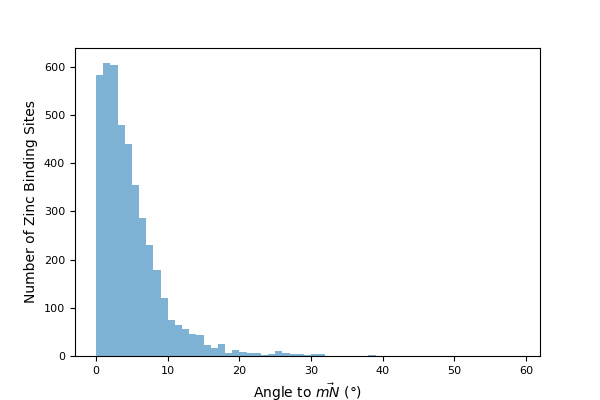

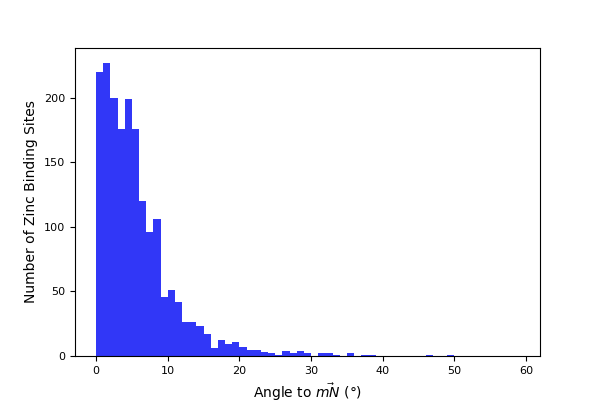


**a)**

**Figure S10: Distributions of two types of angles (in degrees) between predicted/resolved zinc ions and histidine imidazole groups within predicted and resolved zinc-binding sites.** The histograms illustrate two key angles: Angle_to_Plane (α) (a, b) and Angle_to_$\vec{mN}$ (β) (c, d), used for guiding zinc ion placement through the incorporation of ideal constraints on coordination bond angles, based on established data [3]. These angles, defined in Section 3.1.3 and depicted in Figure 8, were measured across zinc-binding sites in the training set. Angle_to_Plane (α) is the angle between the coordination bond vector and the imidazole plane, while Angle_to_$\vec{mN}$ (β) represents the angle between the coordination bond vector and the vector from the midpoint of imidazole carbons to the nitrogen atom coordinating the zinc ion. Two distributions are analyzed for each angle: (i) Panels (a) and (c), show the distributions for correctly predicted zinc-binding sites, encompassing all binding residues and their predicted zinc ion positions. (ii) Panels (b) and (d) correspond to resolved zinc-binding sites from PDB structures. The high similarity between the predicted and resolved distributions, validats the use of ideal angle constraints for zinc ion positioning, and demonstrates ZincSight's accuracy in reproducing experimentally determined preferences for coordination bond angles within zinc-binding sites.


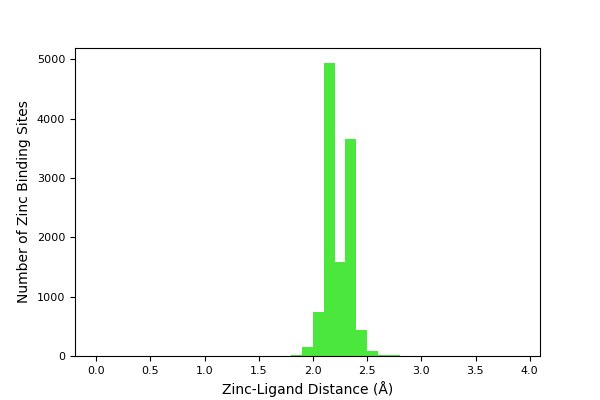

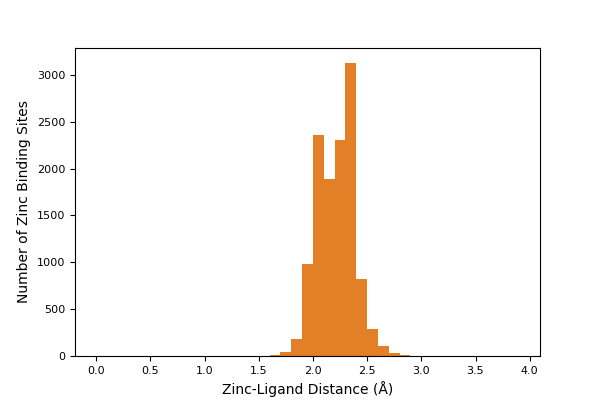


**a)**

**b)**

**Figure S11: Distribution of zinc-ligand distances (Å) in predicted and resolved zinc-binding sites.** The histograms present the distributions of distances (Å) between predicted/resolved zinc ions and their binding ligands within both predicted (a) and resolved (b) zinc-binding sites in the training set. Predicted sites are those that include all actual binding residues. ZincSight utilizes specified optimal zinc-ligand coordination bond lengths to position zinc ions within candidate sites. These distances are based on available data [2], [4]. The majority of measured zinc-ligand distances fall within 2.0 Å to 2.5 Å for both predicted and resolved sites. The similarity consistency validates ZincSight's zinc ion positioning approach and confirms the suitability of the selected ideal zinc-ligand.


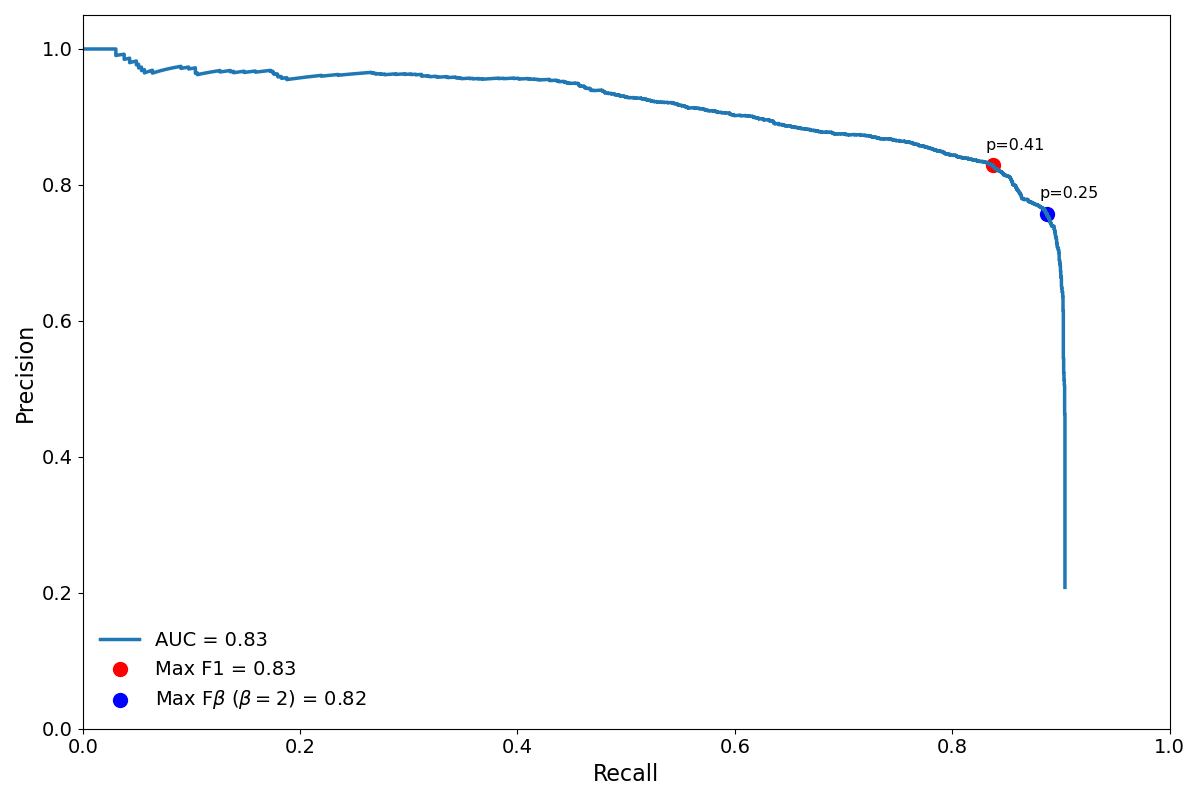

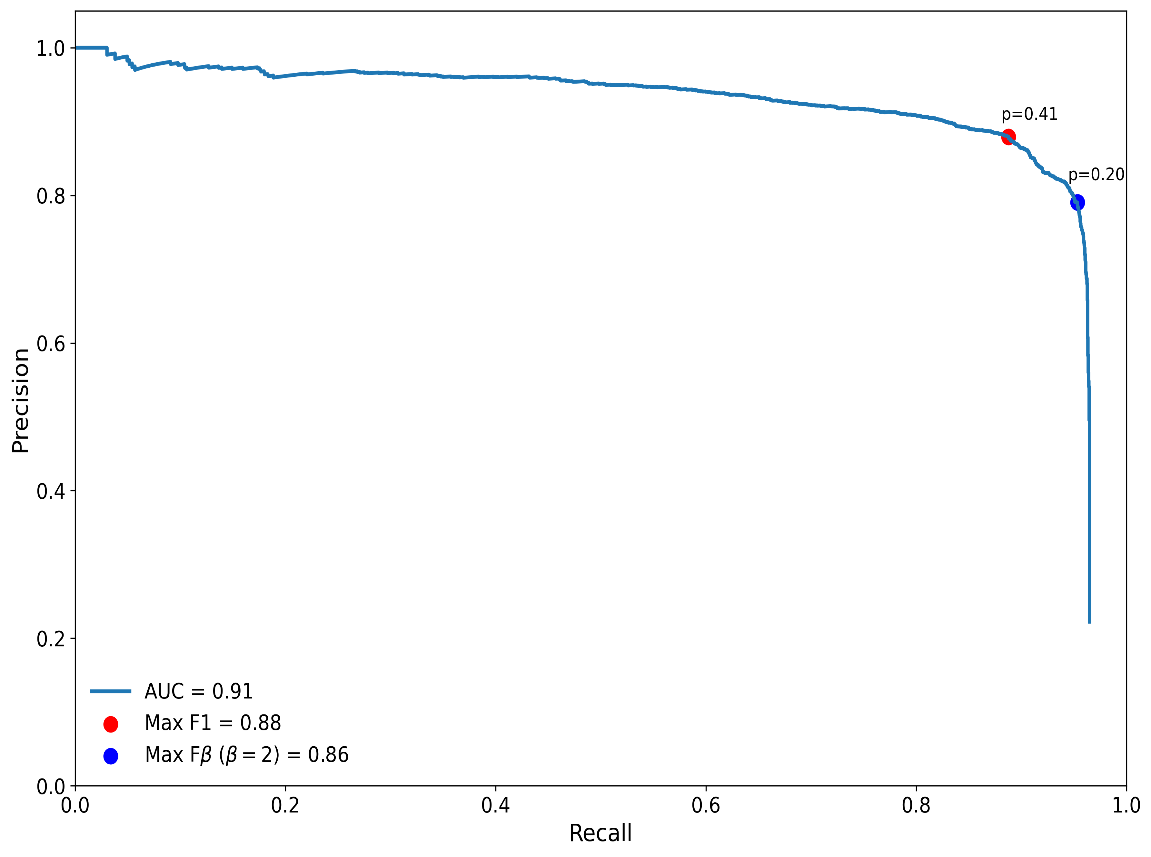


**b)**

**a)**

**Figure S12: Identification of zinc-binding sites' residues in the training set.** Precision-Recall curve for ZincSight prediction task for all zinc-binding sites within 2043 protein structures in the training set. (a) TP predictions are defined as predicted binding sites where at least three predicted binding residues match the experimentally verified binding residues, and all predicted residues are involved in zinc-binding. FP predictions are defined as cases where fewer than three of the predicted binding residues are involved in zinc-binding, or when not all predicted binding residues at the same site are actually involved in binding. For example, a prediction with four binding residues where only three of the predicted residues bind zinc. (b) TP predictions are defined as predicted binding sites where all predicted binding residues match the experimentally verified binding residues. FP predictions are defined as cases where not all experimentally verified binding residues are included within the prediction, or when not all predicted binding residues are involved in zinc-binding. Maximal F1 and Fβ (β=2) scores, along with their corresponding ZincSight scores, are annotated for both curves.


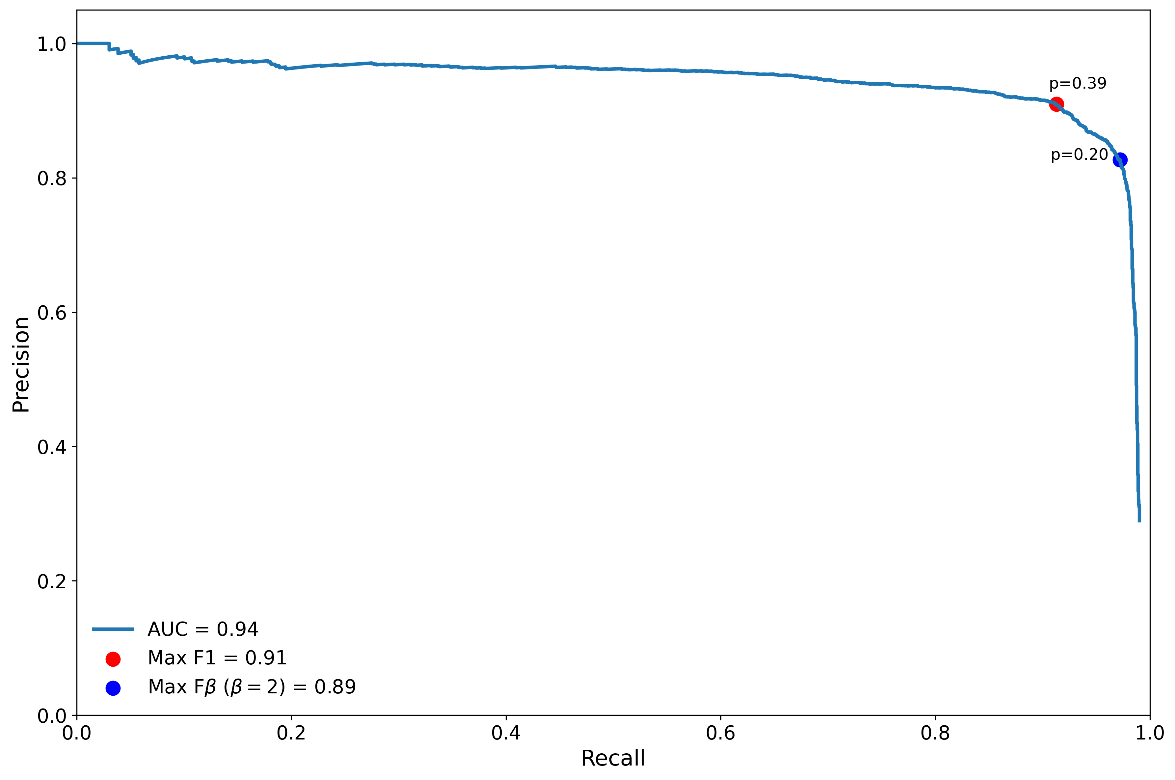


**Figure S13: Identification of zinc-binding sites within 5 Å in the training set.** Precision-Recall curve for ZincSight prediction task for all “3+ zinc-binding sites” within 2043 protein structures within the training set. TP predictions are defined as predicted sites within 5 Å of an experimentally verified zinc site. FP predictions within a 5 Å radius were clustered and counted as a single instance per cluster. Maximal F1 and Fβ (β=2) scores, along with their corresponding ZincSight scores, are annotated on the curve.

**
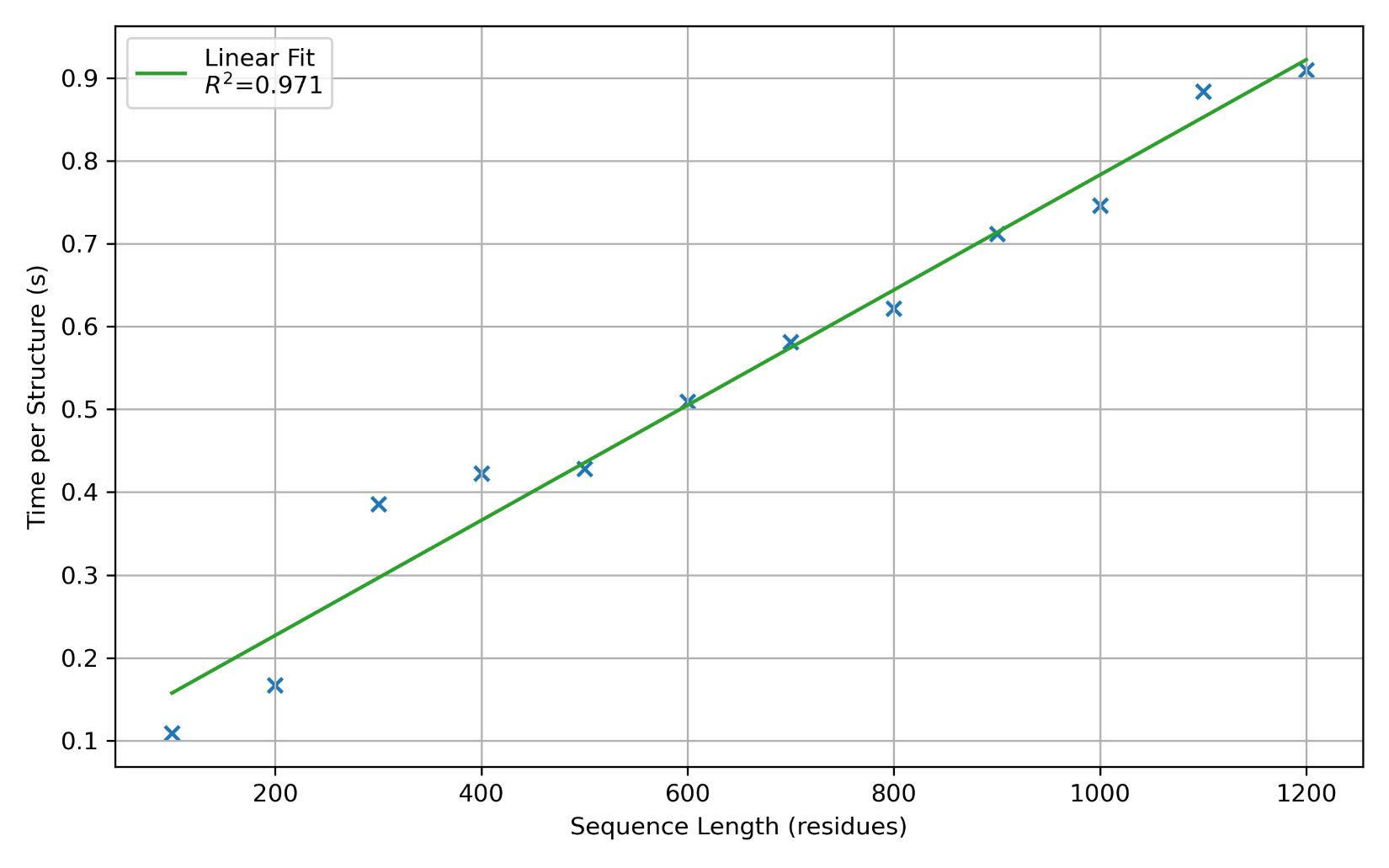
**

**Figure S14: Run-time scaling with protein size (residue number).** A scatter plot depicting computational run times for batches of 300 proteins of varying sequence lengths. The evaluated structures are AF2-predicted protein models, randomly selected from UniProt queries based on length categories indicated by labeled ticks, each encompassing a range of $\pm1$ residues around the specified length. A linear regression fit to the data yields a coefficient of determination ($R^{2}$) of 0.971, demonstrating that run-time scales linearly with protein length within the tested range.

## References

[1] T. Dudev and C. Lim, “Competition among Metal Ions for Protein Binding Sites: Determinants of Metal Ion Selectivity in Proteins,” *Chem. Rev.*, vol. 114, no. 1, pp. 538–556, Jan. 2014, doi: 10.1021/cr4004665.

[2] M. Laitaoja, J. Valjakka, and J. Jänis, “Zinc Coordination Spheres in Protein Structures,” *Inorg. Chem.*, vol. 52, no. 19, pp. 10983–10991, Oct. 2013, doi: 10.1021/ic401072d.

[3] P. Chakrabarti, “Geometry of interaction of metal ions with histidine residues in protein structures,” *Protein Eng Des Sel*, vol. 4, no. 1, pp. 57–63, 1990, doi: 10.1093/protein/4.1.57.

[4] B. Tamames, S. F. Sousa, J. Tamames, P. A. Fernandes, and M. J. Ramos, “Analysis of zinc‐ligand bond lengths in metalloproteins: Trends and patterns,” *Proteins*, vol. 69, no. 3, pp. 466–475, Nov. 2007, doi: 10.1002/prot.21536.

[5] Platt, John and others, “Probabilistic outputs for support vector machines and comparison to regularized likelihood methods,” *Advances in Large Margin Classifiers*, vol. 10, no. 3, pp. 61–74, 1999.

[6] G. W. Brier, “Verification of Forecasts Expressed in Terms of Probability,” *Mon. Wea. Rev.*, vol. 78, no. 1, pp. 1–3, Jan. 1950, doi: 10.1175/1520-0493(1950)078<0001:VOFEIT>2.0.CO;2.
